# Ternary heterostructure-driven photoinduced electron-hole separation enhanced oxidative stress for triple-negative breast cancer therapy

**DOI:** 10.1186/s12951-024-02530-4

**Published:** 2024-05-12

**Authors:** Shuqing Dong, Yuqi Huang, Hanrong Yan, Huarong Tan, Liying Fan, Minghao Chao, Yiping Ren, Ming Guan, Jiaxin Zhang, Zhao Liu, Fenglei Gao

**Affiliations:** 1 Department of Laboratory Medicine, Shanghai Medical College, Huashan Hospital, Fudan University, Shanghai, 200040 China; 2 Jiangsu Key Laboratory of New Drug Research and Clinical Pharmacy, Xuzhou Medical University, Xuzhou, 221004 China; 3 Department of Thyroid and Breast Surgery, Affiliated Hospital of Xuzhou Medical University, Xuzhou, 221004 China

**Keywords:** Plasmon resonance, Oxidative stress, Ternary heterostructures, e^−^-h^+^ pairs

## Abstract

Zinc oxide nanoparticles (ZnO NPs) stand as among the most significant metal oxide nanoparticles in trigger the formation of reactive oxygen species (ROS) and induce apoptosis. Nevertheless, the utilization of ZnO NPs has been limited by the shallowness of short-wavelength light and the constrained production of ROS. To overcome these limitations, a strategy involves achieving a red shift towards the near-infrared (NIR) light spectrum, promoting the separation and restraining the recombination of electron-hole (e^−^-h^+^) pairs. Herein, the hybrid plasmonic system Au@ZnO (AZ) with graphene quantum dots (GQDs) doping (AZG) nano heterostructures is rationally designed for optimal NIR-driven cancer treatment. Significantly, a multifold increase in ROS generation can be achieved through the following creative initiatives: (i) plasmonic Au nanorods expands the photocatalytic capabilities of AZG into the NIR domain, offering a foundation for NIR-induced ROS generation for clinical utilization; (ii) elaborate design of mesoporous core-shell AZ structures facilitates the redistribution of electron-hole pairs; (iii) the incorporation GQDs in mesoporous structure could efficiently restrain the recombination of the e^−^-h^+^ pairs; (iv) Modification of hyaluronic acid (HA) can enhance CD44 receptor mediated targeted triple-negative breast cancer (TNBC). In addition, the introduced Au NRs present as catalysts for enhancing photothermal therapy (PTT), effectively inducing apoptosis in tumor cells. The resulting HA-modified AZG (AZGH) exhibits efficient hot electron injection and e^−^-h^+^ separation, affording unparalleled convenience for ROS production and enabling NIR-induced PDT for the cancer treanment. As a result, our well-designed mesoporous core-shell AZGH hybrid as photosensitizers can exhibit excellent PDT efficacy.

**Supplementary Information:**

The online version contains supplementary material available at 10.1186/s12951-024-02530-4.

## Introduction

Within mitochondria and peroxisomes, reactive oxygen species (ROS) have assumed pivotal roles in numerous signaling pathways, finely orchestrating physiological and pathological functions [1, 2]. In cancerous tissues, ROS exhibit a proximate correlation with tumorigenesis and facilitates the progression of tumors; however, it has been experimentally demonstrated that elevated ROS levels manifest cytotoxic effects, leading to the demise of neoplastic cells [3, 4]. This heightened ROS functionality primarily arises from oncogenic receptor activity. Excessive ROS levels can instigate irrevocable harm to intracellular constituents, encompassing organelles and the cytoskeleton, ultimately culminating in the demise of tumor cells [5, 6]. Non-invasive therapeutic approaches, notably the field of photodynamic therapy (PDT), have garnered significant attention, primarily due to their capacity for precise spatiotemporal control and their minimal propensity for adverse effects, as extensively studied [7–9]. In the realm of nanomaterials, such as quantum dots (QDs) [10, 11], zinc oxide nanoparticles (ZnO NPs) [12], silicon (Si), and titanium dioxide (TiO_2_) [13, 14], their potential as nano photosensitizers has attracted interest for their remarkable photodegradation resistance.

ZnO NPs represent essential semiconductor materials possessing intriguing photoresponsive characteristics [15]. Over time, ZnO-based NPs have exhibited considerable potential in the realm of PDT owing to their biocompatibility and remarkable ability to generate tumor-destructive ROS via mild photodynamic activation [16–19]. In the realm of oncological research, ZnO NPs manifest a distinct proclivity for inducing cellular toxicity specific to cancer cells. This propensity is attributed to their remarkable capacity to engender ROS and disrupt the structural integrity of the mitochondrial membrane [20–23]. While the potential of ZnO NPs in precisely targeting malignant cells has garnered considerable attention, it remains imperative to acknowledge the extant challenges that confront their practical application. These challenges encompass the swift recombination of e^−^-h^+^ pairs, which necessitates UV light for activation [24]. In general, accelerated charge recombination often leads to a constrained generation of ROS, thereby hindering the efficacy of PDT. The limited responsiveness of ZnO NPs exclusively to UV radiation serves to constrain their utility in the treatment of deeply situated tumors [25]. This constraint stems from the inherent incapacity of UV light to effectively permeate deep tissue regions, as it is predominantly absorbed by the adjacent biological milieu.

Plasmonic metallic nanostructures, as exemplified by gold nanorods (Au NRs), offer an avenue for tailored enhancement of photocatalytic performance in semiconductors, particularly within the visible and NIR spectral regions, thereby augmenting the scope for clinical applications by extending the depth of penetration [26–28]. Consequently, a plethora of methodologies have been devised to amalgamate the distinctive surface plasmon resonance (SPR) inherent to Au NRs with ZnO (Zhou et al., 2021), employing core-shell architectures and coating techniques [29, 30]. Moreover, amalgamating plasmonic Au NRs with ZnO within a precisely engineered core-shell nanoarchitecture holds the promise of enhancing charge carrier excitation and transfer, culminating in a substantial enhancement in the efficacy of photocatalytic procedures. Nevertheless, the ROS yield of this composite system, denoted as Au@ZnO (AZ), remained constrained. The conventional core-shell configuration and coating approach fail to effectively segregate the spatial distribution of energetic e^−^-h^+^ pairs [31].

On the other hand, graphene’s incorporation as a co-catalyst has sparked considerable interest due to its distinct attributes, including high thermal conductivity, exceptional charge carrier mobility, expansive surface area, and mechanical stability [32, 33]. As a co-catalyst, graphene offers notable advantages: it provides a robust scaffold for anchoring finely dispersed metallic or oxide nanoparticles, acts as a highly conductive matrix for efficient electrical contact, facilitates electron transfer from the semiconductor’s conduction band, enhancing charge separation efficiency, and serves as a co-catalyst for ROS generation, courtesy of its extensive surface area and electron mobility [34, 35]. Hence, to enable NIR radiation-triggered PDT, the design of graphene doping strategies becomes crucial. These strategies must effectively extend the separation of hot e^−^-h^+^ pairs, ultimately promoting increased ROS production.

Photothermal therapy (PTT), a highly promising approach for cancer treatment, has garnered significant attention due to its favorable treatment outcomes. PTT agents, concentrated within tumors, can effectively engage with external laser sources, generating localized heat to eradicate tumors while minimizing damage to adjacent healthy tissue [36]. Au NRs, recognized as exemplary PTT agents, exhibit robust light absorption in the NIR region, showcasing remarkable therapeutic efficacy in PTT applications.

**Scheme 1:**
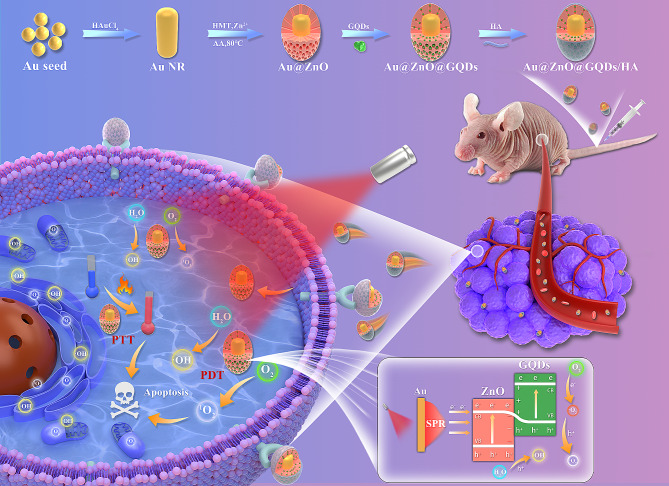
Schematic diagram of the synthesis of AZGH NPs and the therapeutic process of TNBC

In this study, we propose a novel approach utilizing GQDs incorporated within mesoporous AZ NPs, further functionalized with hyaluronic acid (HA). Within this framework, plasmonic Au NRs serve as an energy source, effectively generating ROS at the tumor site. Moreover, the introduced Au NRs emerge as highly promising catalysts for facilitating PTT, a modality demonstrated to proficiently kill tumor cells through the induction of apoptosis. ZnO, known for its capability to accept high-energy electrons, complements this system. Furthermore, the introduction of GQDs as dopants acts as a barrier, effectively preventing the rapid recombination of e^−^-h^+^ pairs. Consequently, our designed Au@ZnO@GQDs/HA (AZGH) NPs exhibit the potential to significantly enhance PDT efficiency by mitigating e^−^-h^+^ pairs recombination, all while offering an approach for the therapy associated with triple-negative breast cancer (TNBC). This innovative design we present here represents a promising avenue to elevate the efficacy of PDT, thus contributing to its broader clinical application (Scheme 1).

## Experimental

### Synthesis of AuNR@ZnO

AuNR@ZnO core/shell nanoparticles involved the the Zn^2+^ precursors hydrolysis in an alkaline milieu [37]. In brief, a solution was created by gently combining 24 mM CTAB, 12 mM AA, 24 mM freshly prepared Zn(NO_3_)_2_, and 24 mM HMT, each in equal parts, which served as the growth solution. Subsequently, 4.0 mL CTAB-stabilized Au NRs underwent two washes via centrifugation employing deionized water. Following this, the aforementioned growth medium (12.0 mL) was added to a specific quantity of Au NRs, confined within a glass receptacle. The Zn^2+^/Au NRs volume ratio was maintained at 3:7. The pH of the resulting amalgam was titrated to 9.0 through the addition of a 0.1 M NaOH solution. The amalgamation was subsequently exposed to an 80 ℃ convection oven, where it remained tranquil for a duration of 6 h. Finally, the synthesized AuNR@ZnO nanoparticles were purified through a process of washing with deionized water and centrifugation at 8000 rpm.

### Synthesis of AuNR@ZnO@GQDs

In the as-synthesized AuNR@ZnO, immersion took place within a specified volume of GQDs stock solution, accompanied by vigorous magnetic agitation. The resulting blend was stirred for a period of 5 min, followed by centrifugation at a velocity of 8000 revolutions per min for a period of 300 s.

### Synthesis of AuNR@ZnO@GQDs-HA

A 25 mL aliquot of deionized water was meticulously introduced to HA (100 mg), with the HA achieving complete solubilization via ultrasonic treatment. Subsequently, the resulting AuNR@ZnO@GQDs composite was introduced into the HA solution and subjected to vigorous agitation under ambient conditions for a duration of 24 h. Following this agitation period, the ultimate product was acquired through centrifugation [38].

### ^1^O_2_ detection in vitro

To substantiate the production of ^1^O_2_, the singlet oxygen sensor green (SOSG) probe had been employed. 2 mL AZGH suspension (100 μg mL^− 1^) was amalgamated within a 2 μM pre-prepared stock solution and subsequently subjected to irradiation using an 808 nm laser over varying time intervals. The resulting time-dependent fluorescence, emanating from the oxidation of SOSG, was qualitatively evaluated by means of a fluorescence microplate reader.

### •OH generation in vitro

To elucidate the generation of •OH species facilitated by AZGH nanoparticles, we harnessed methylene blue (MB) as a reagent. The oxidative prowess of •OH was employed to prompt the alteration in absorption at 665 nm in MB. 2 mL aliquot of AZGH (100 μg mL^− 1^) was conjoined within a buffer solution containing MB (10 μg mL^− 1^), subjected to irradiation using an 808 nm laser with variable exposure durations. Subsequently, the reaction transpired under constant conditions at 37 °C. To probe the optical properties of the solution, an absorbance measurement was performed at a specific wavelength of 665 nm.

### •OH and ^1^O_2_ evaluation by ESR

At ambient temperature, the generation of •OH and ^1^O_2_ species was efficiently trapped through the utilization of an ESR instrument. Specifically, the quantification of ^1^O_2_ and •OH was judiciously accomplished by employing TEMP and DMPO. To initiate the experiments, a combination of 50 mL of AZ solution (100 μg mL^− 1^), 10 mL each of DMPO and TEMP was meticulously prepared. The same rigorous protocol was consistently followed for AZG (100 μg mL^− 1^). The reaction mixture was contained within a quartz capillary, rendering its suitability for subsequent analysis. For all NIR groups, the compound underwent exposure to an 808 nm laser operating at 1.0 W cm^− 2^ for a duration of 10 min prior to detection.

### Intracellular uptake analysis

In order to scrutinize the intracellular uptake of AZGH, a Petri dish (35 mm) was used to plate 4T1 cells (positive CD44 expression) and L929 cells (negative CD44 expression), with each dish containing either 5 × 10^4^ cells through a 12 h incubation period. Subsequently, these cells were exposed to a culture medium consisting of 1.5 mL of RPMI-1640 solution, incorporating either 100 μg mL^− 1^ of AZGH or AZG. The incubation was conducted for durations of 1, 4, and 8 h at a constant temperature, respectively. Following this exposure, the cells underwent a triple wash with PBS and were subjected to nuclear staining with DAPI for a duration of 5 min, after which they were imaged using a CLSM.

### Intracellular ROS detection

Five experimental groups, namely the control group (PBS), NIRgroup, AZ + NIR group, AZG + NIR group, and AZGH + NIR group, were established to investigate and compare the in vitro efficiency of ROS production. The intracellular ROS detection was systematically executed via CLSM, and DCFH-DA was greatly employed to facilitate this endeavor as a ROS fluorescent marker probe. 4T1 cells were subjected to an incubation period with disparate reagents in tandem with DCFH-DA (20 μM) for a stipulated temporal span of 40 min. Consecutively, the cells were subjected to irradiation via an 808 nm laser (10 min, 1 W cm^− 2^). ROS generation was observed and documented via the application of confocal laser scanning microscopy and flow cytometer.

### In vitro anticancer efficacy

4T1 cells were plated in 96-well microplates and left incubate overnight. Subsequent treatments encompassed different regimens, including (1) Control (PBS), (2) NIR, (3) AZ + NIR, (4) AZG + NIR, and (5) AZGH + NIR, and the CCK-8 assay, as described previously, was employed to assess cell viability. In order to take stock of treatment effect of these diverse therapies, Calcein-AM and PI were subjected to co-staining with cells, for a duration of 50 min. Post-staining, the cells underwent triple rinsing with PBS and were subsequently visualized through the utilization of an inverted fluorescence microscope. Moreover, to quantify cellular apoptosis, flow cytometry was employed. Following 48 h incubation period, cell cultures were cleaned with PBS, followed by a 15 min staining procedure with Annexin V/propidium iodide. Ultimately, the cell apoptosis was detected by flow cytometry, Ultimately, flow cytometry was employed to detect cell apoptosis. After that, the resultant data was subjected to analysis using FlowJo software.

### Animals

In the context of in vivo research, Balb/c nude mice, approximately 5 weeks old, were procured from Gempharmatech Co., Ltd. All animal-handling protocols abided by the ethical and scientific criteria stipulated and endorsed by the Experimental Animal Ethics Committee of Xuzhou Medical University. The construction of the 4T1 female nude mice tumor model was executed by injecting on the right shoulder subcutaneous by means of approximately 2 × 10^6^ 4T1 cells, inclusived the 120 μL PBS. Subsequently, these mice were incorporated into the ensuing experimental protocols, once the volume (computing method: (length×(width)^2^)/2) attained approximated 100 mm^3^.

### Hemolysis assay

In order to ascertain the potential hemolytic effects of AZGH NPs, we introduced a 0.3 mL erythrocyte suspension from Balb/C nude mice into three distinct solutions. These solutions inclusived 1 mL deionized water, serving as the positive control, and 1 mL PBS, representing the negative control. Additionally, separate AZGH NPs dispersions were provided for varying concentrations, ranging from 12.5, 25, 50, 75, and 100 μg mL^− 1^. Subsequently, these mixtures were subjected to an incubation period of 12 h at 37 °C, after which the absorbance of each specimen was assessed at a wavelength of 540 nm.

### In vivo laser induced thermal imaging

IRT, standing for infrared thermal imaging, was adopted through the utilization of an IR thermal camera, while employing an excitation laser tuned to a wavelength of 808 nm. Upon the attainment of a tumor size of approximately 100 mm³, the tumor sites encompassed three distinct cohorts, for instance, (1) mice injected solely with PBS; (2) mice subjected to intravenous administration of AZG (100 μg mL^− 1^); (3) mice receiving intravenous injection of AZGH (100 μg mL^− 1^). Subsequent to that, the location of tumor was exposed to NIR laser irradiation (1.0 W cm^− 2^, 10 min). Real-time imaging and data recording were carried out at various time points.

### In vivo fluorescence imaging

Upon the attainment of a tumor size of approximately 100 mm³, AZGH NPs (100 ml, 5 mg kg^− 1^) were administered via tail injection to BALB/c nude mice. Subsequently, the IVIS Lumina S5 Imaging System was used to perform whole-body fluorescent imaging in vivo at 2, 4, 8, 16, and 24 h intervals subsequent to injection. Post 48 h following the injection, the mice were euthanized in accordance with ethical guidelines. Both tumors and vital organs were excised for the purpose of in vivo fluorescence imaging to scrutinize the distribution of nanoparticles within the tissues.

### In vivo anticancer treatment performance

Upon reaching a tumor volume of approximately 100 mm³, the therapeutic potential of multimodal oncotherapy was investigated. In accordance with the experimental design, mice were apportioned into one of five cohorts (*n* = 4) at random, as delineated below: (1) Control (PBS); (2) NIR; (3) AZ + NIR; (4) AZG + NIR; (5) AZGH + NIR. A uniform dosage of the test agents (100 μg mL^-1^, 200 μL) was administered to all mice via the tail vein. Following a 24 h post-injection interval, each cohort underwent irradiation employing NIR laser, delivering a radiant power of 1.0 W cm^-2^; for a duration of 10 min. The progression of the treatment protocol was meticulously monitored through daily assessments of mice weights, dimensions, and tumor volumes throughout the duration of the study. Upon conclusion of the treatment regimen, marking the 14th day, all mice were dealt by the humane euthanization. Subsequently, the harvested tumors underwent a comprehensive assessment, encompassing photography, weight measurement, and thereafter, histological inspection through the application of hematoxylin and eosin (H&E) staining.

### Statistical analysis

All experimental results were presented in the form of mean values ± S.D. Statistical comparisons were conducted using a one-way analysis of variance (ANOVA). ***p < 0.001, **p < 0.01, or *p < 0.05.

## Results and discussion

### Preparation and characterization of the AZGH NPs

**Fig. 1:**
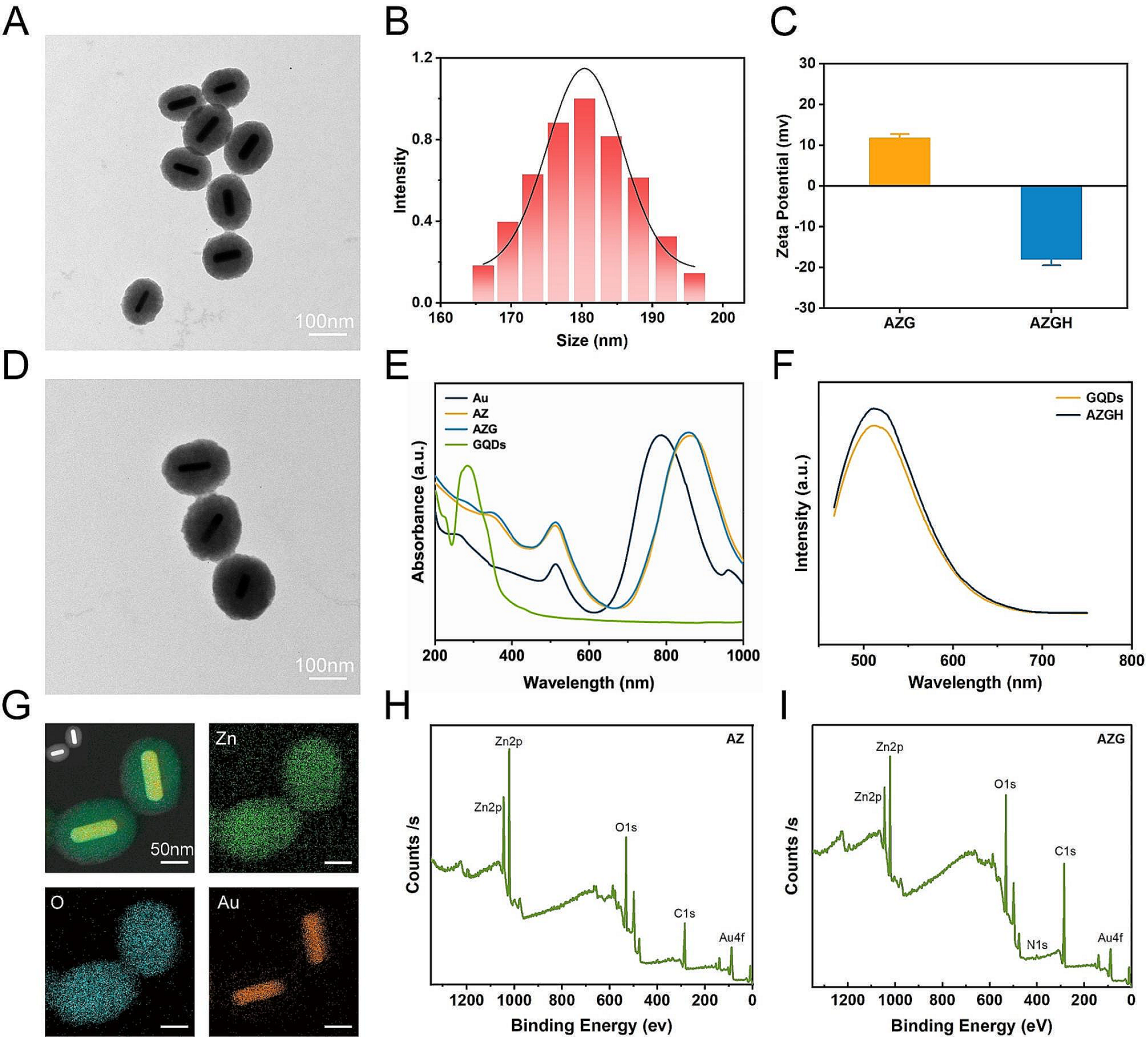
Establishment and characterization of AZGH NPs. (**A**) TEM image of AZ NPs (Scale bar: 100 nm). (**B**) The diameters distribution curve of the AZGH NPs. (**C**) Zeta potential of the AZG and AZGH NPs. (**D**) TEM image of AZGH NPs (Scale bar: 100 nm). (**E**) UV-vis spectroscopy of Au, AZ, AZG and GQDs NPs. (**F**) The PL spectra of GQDs and AZGH NPs. (**G**) EDX element mapping of AZGH NPs. (**H**) XPS spectrum of AZ NPs. (**H**) XPS spectrum of AZG NPs

Mesoporous core-shell heterostructures of AZGH were successfully fabricated employing a systematic four-step technique. Precisely, uniform Au NRs were synthesized with an average aspect ratio of approximately 7:1 (Figure S1A) [39]. AZ were synthesized through an ascorbic acid-assisted growth approach, building upon previous design with certain refinements [37]. Following this, uniform AZ core-shell nanostructures was achieved by means of the deposition of a mesoporous ZnO layer around the Au NRs (Figure S1B). Figure S1C shows the typical HRTEM image of GQDs. The features of GQDs were elucidated in Figure S2. UV illumination at 365 nm, GQDs suspended in water exhibit vibrant green fluorescence [40]. The PL spectra of GQDs exhibit a gradual increase at 515 nm, whenthe concentration varies within the range of 12.5 to 200 μg mL^− 1^. It is worth noting that the PL spectra of AZGH reveal analogous emission peaks to those observed with GQDs, providing initial evidence substantiating the successful doping of GQDs into AZGH (Fig. 1F). Subsequently, the formation of AZG ensued through the rapid injection of a well-suited mixture of GQDs into the mesoporous core-shell nanostructures of AZ at ambient temperature (Fig. 1A). The final step involved the loading of the targeting agent HA onto AZG to yield AZGH NPs, which exhibited a distinctive elliptical configuration, as showed by the TEM images (Fig. 1D), boasting an average diameter of around 180 nm (Fig. 1B). The AZGH NPs achieve homogeneous dispersion under physiological conditions, maintaining their average size virtually unaltered over a 24 h duration (Figure S3), which is of paramount importance for biological applications. Initial validation of AZGH NPs were investigated utilizing UV–vis spectroscopy (Fig. 1E). The absorption peak pertaining to Au nanorods is conspicuously situated at approximately 790 nm. Notably, the UV-vis extinction spectra of AZ NPs reveal a prominent redshift in the longitudinal SPR bands when compared to their Au NRs counterparts. A discernible peak emerged at 352 nm, signifying that the surface of the Au NRs was successfully encased with the formation of a ZnO shell structure. The UV-vis spectra of AZG NPs exhibit a prominent peak at around 280 nm, a distinctive attribute attributed to the presence of GQDs. The investigation of the electronic structure and chemical composition about AZG NPs was undertaken through the application of XPS. Presented in Fig. 1I were the high-resolution XPS spectra delineating the core levels of Au (4f), Zn (2p), C (1s), and O (1s) species encompassed within the AZG NPs, while AZ NPs were Presented in Fig. 1H. Additionally, Figure S4A showcases the four core levels of both Au and ZnO. Notably, the prominent spectral features were discerned at binding energies of 88.8 and 86.7 electronvolts (eV), which amount to the core levels of the Au 4f_7/2_ and Au 4f_5/2_ states, denoting a zero valence state (Au^0^) [41]. Conversely, the distinctive features at elevated binding energies, namely 92.2 and 90.3 eV, ascribed to the peaks of the Zn 3p_3/2_ and Zn 3p_1/2_ core levels, signifying a valence state of Zn^2+^ [42]. In Figure S4B, a pair of distinctive features manifests itself at binding energies of 1045.0 and 1021.9 eV, representating Zn 2p_3/2_ and Zn 2p_1/2_ states, respectively [43]. Furthermore, a couple of appreciably heightened signal, notably observed in the C = O and C-O-C domains, within the AZG NPs hints at the binding of C in graphene and O in ZnO (Figure S4C). Notably, the binding energies pertaining to O1 at 531.3 eV, exhibit an overlap and are attributed to the presence of C = O and C-O chemical bonds, whereas O2 and O3, residing within the 532.1-533.1 eV range, are ascribed to the existence of C-OH [44]. Collectively, those compelling body of consequence further demonstrates that C element from GQDs is conjoined with O element from ZnO (Figure S4D). To analyze the composition of the AZGH NPs, an elemental mapping of AZGH NPs was conducted. The findings unequivocally reveal that the AZGH NPs predominantly comprise elements from the Au, O, and Zn components (Fig. 1G and Figure S5). Fourier transform infrared spectroscopy (FT-IR) further corroborated the successful synthesis of AZGH NPs. As shown in Figure S6, the absorption peaks at approximately 3350 cm^− 1^ were assigned to O − H and *N* − H stretching vibrations from HA. The usual glycosyl peaks in the HA structure (1150 cm^− 1^) also indicates the presence of HA. Additionally, in comparison with AZG NPs, the zeta potentials of AZGH NPs exhibited negativity, thus confirming the effective loading of HA onto their surface (Fig. 1C).

### ROS generation properties and photothermal properties

**Fig. 2:**
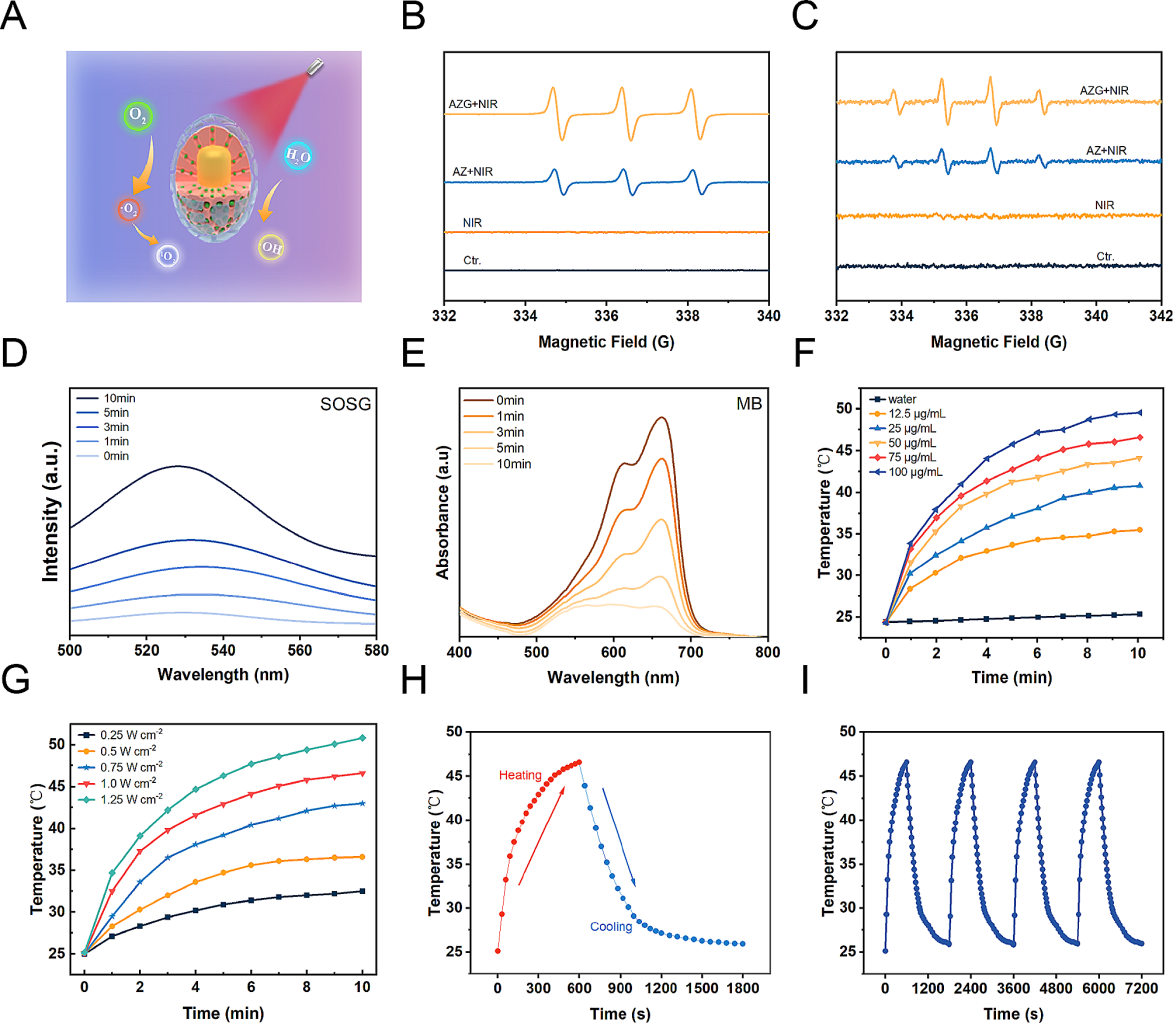
Illustration of ROS generation and o photothermal effect in AZGH NPs. (**A**) A schematic illustration of ROS generation. (**B**) ESR spectra of ^1^O_2_ trapped by TEMP under different groups. (**C**) ESR spectra of •OH trapped by DMPO under different groups. (**D**) Generation of ^1^O_2_ at different times measured using SOSG as the probe. (**E**) Production of •OH at different times using MB as the probe. (**F**) Photothermal curves of AZGH NPs aqueous solutions at various concentrations (0, 12.5, 25, 50, 75, and 100 μg mL^− 1^) under the NIR irradiation (808 nm, 1.0 W cm^− 2^) for 10 min. (**G**) Photothermal curves of 75 μg mL^− 1^ of aqueous AZGH NPs irradiated with NIR laser (808 nm, 0.25, 0.5, 0.75, 1.0, and 1.25 W cm^− 2^) for 10 min. (**H**) Heating and cooling curve of an aqueous dispersion of AZGH NPs at 75 μg mL^− 1^ concentration under 808 nm laser irradiation (1.0 W cm^− 2^). (**I**) Recycling-heating profiles of AZGH NPs aqueous solution at 75 μg mL^− 1^ concentration after 808 nm laser irradiation at 1.0 W cm^− 2^ for four laser on/off cycles

The crucial role of GQDs within the context of AZGH NPs involves facilitating the transfer of photoexcited electrons from Au NRs upon exposure to NIR radiation to ZnO. This efficient electron transfer process effectively impedes the recombination of e^−^-h^+^ pairs, leading to the substantial generation of ROS, including •OH and ^1^O_2_ (Fig. 2A). Photoluminescence (PL) emission spectroscopy stands as a potent tool for elucidating the radiative recombination dynamics of photoexcited carriers within photocatalysts. Broadly speaking, a diminished recombination rate of photoexcited electron-hole pairs corresponds to a lower PL emission intensity. In this context, we conducted PL spectral analyses on both AZ and AZG. As depicted in Figure S7, the PL intensity of AZG experiences a noteworthy reduction compared to that of AZ. This observation implies that GQDs effectively impede the recombination of photoexcited e^−^-h^+^ pairs in AZ, consequently contributing to a further enhancement in photocatalytic performance. The ROS generation was investigated via electron spin resonance (ESR) spectroscopy, while 5,5-dimethyl-1-pyrroline N-oxide (DMPO) functions as a •OH trapping agent, and 2,2,6,6-tetramethyl-4-piperidine (TEMP) serves as a ^1^O_2_ trapping agent [45]. As depicted in Fig. 2B, “AZ + NIR” yielded a representative 1:1:1 triplet ESR signal, thereby confirming the generation of TEMP/^1^O_2_ adduct. The other way around, the “AZG + NIR” group exhibited a more robust ESR signal intensity, suggesting enhanced ^1^O_2_ generation possibly attributable to the involvement of GQDs. Similarly, the more businesslike generation of •OH was observed in “AZG + NIR”, showing the highest ESR signal amplitude associated with the DMPO/•OH adduct, displaying a typical 1:2:2:1 quartet ratio (Fig. 2C). Moreover, ^1^O_2_ production of AZG NPs at different time points ranging from 0 to 10 min were monitored under NIR irradiation. Upon prolonged NIR exposure, a notable augmentation in the absorption of SOSG was observed, signifying the generation of ^1^O_2_ during the course of the experiment (Fig. 2D) [46]. The production of •OH by AZG NPs was found to be contingent upon the duration of NIR exposure. As anticipated, the emblematic absorbance peak corresponding to the MB at 664 nm exhibited a decrement along with the extension to the duration of NIR exposure (Fig. 2E) [47]. Subsequently, the assessment of •OH generation was conducted employing 3,3′,5,5′-tetramethylbenzidine (TMB) as the sensor. Initially, TMB underwent oxidation, resulting in the formation of blue oxTMB, which was quantified through spectroscopic analysis. Remarkably, a progressive elevation in the concentration of AZGH NPs led to a corresponding increase in •OH generation (Figure S8). In light of the observation that a pronounced absorption peak within the NIR spectra was manifested in the UV − vis − NIR absorption spectrum of AZG NPs (Fig. 1E), AZGH NPs was designated as a potential NIR photothermal agent. In order to picture the temperature elevation about AZG NPs solution across different concentrations under 808 nm NIR irradiation, infrared thermal imaging techniques were employed (Figure S9). The photothermal heating response subsequently validated the degree of temperature elevation hinging upon the concentration of AZGH NPs, the power intension, and the duration of NIR irradiation (Fig. 2F and G). Importantly, PTT was realized by the time of the temperature raised to ∼45 °C, at which point the concentration was 75 μg mL^− 1^ and the power density was 1 W cm^− 2^. Furthermore, as exemplified in Fig. 2H, the photothermal stabilizing power of the suspension of AZGH NPs (75 μg mL^− 1^) was demonstrated. Notably, even after subjecting the AZGH NPs to 4 cycles of repetitive NIR laser irradiation, the peak temperature remained consistent. These results collectively affirmed the exceptional capability of AZGH NPs in efficiently absorbing and converting NIR light into thermal energy, thereby offering promising prospects for effective PTT treatment (Fig. 2I).

### Safety properties, tumor uptake and ROS production in 4T1 cells

**Fig. 3:**
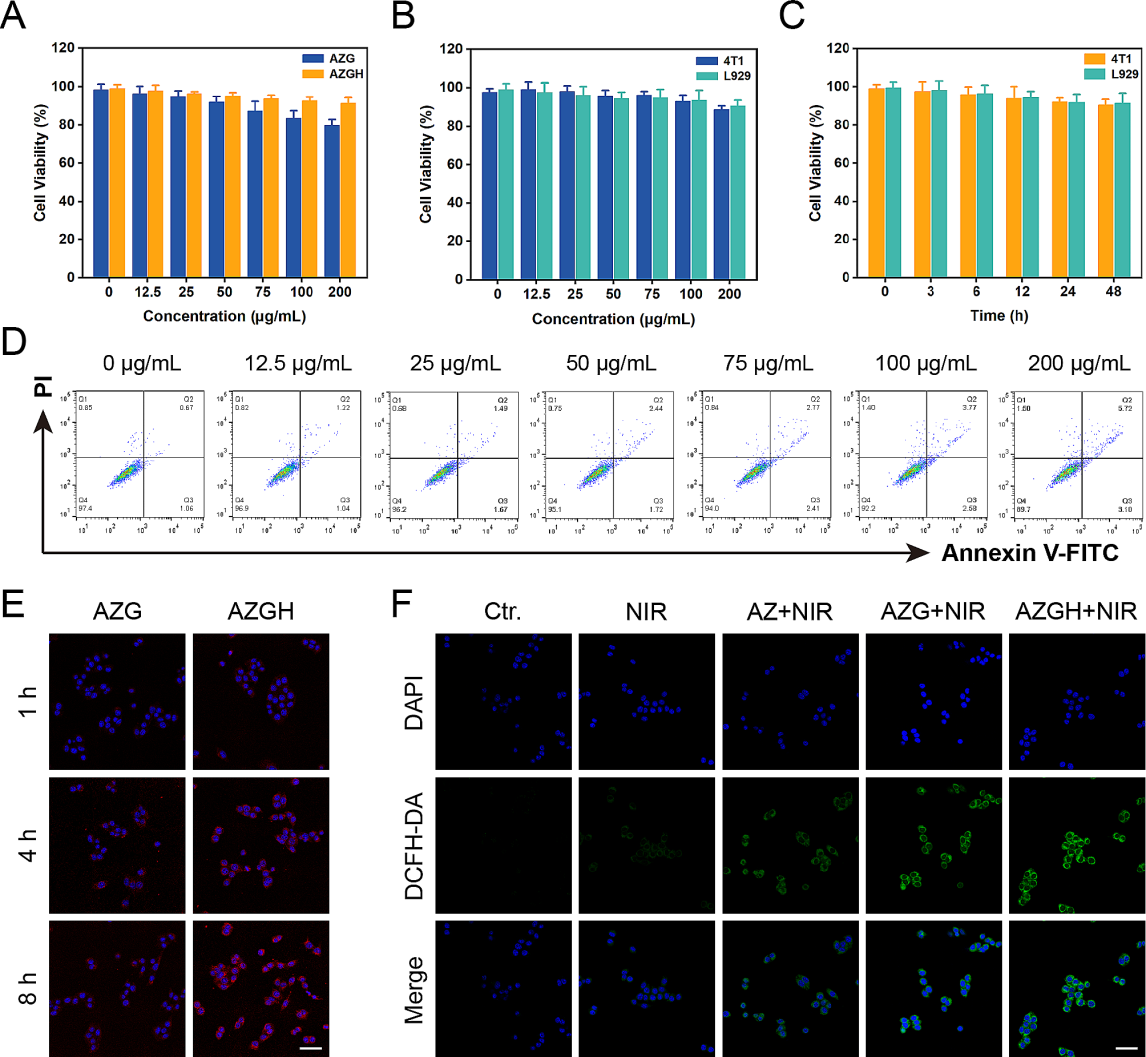
Safety properties, tumor uptake and ROS-generation properties in 4T1 cells. (**A**) Cell viability of 4T1 cells incubated with different concentrations of AZG and AZGH NPs measured by the CCK-8 assay. (**B**) Cell viability of 4T1 and L929 cells after 24 h of co-incubation with different AZGH NPs concentrations. (**C**) Cell viability of 4T1 and L929 cells co-incubation with different incubation times of AZGH NPs. (**D**) Apoptosis/necrosis of 4T1 cells after co-incubation with various dosages of AZGH NPs. (**E**) Fluorescence images of 4T1 cells co-incubation with AZG or AZGH NPs after 1, 4 and 8 h intervention (Scale bar is 50 μm). (**F**) Confocal pictures of 4T1 cells incubated with DCFH-DA after various conditions (Scale bar is 50 μm)

In order to furnish a more thorough assessment of biocompatibility, a CCK-8 evaluation was conducted to elucidate the physiological toxicity about AZGH NPs. The toxic effects of AZGH NPs were assessed by incubating them with 4T1 cells at varying concentrations ranging from 0 to 200 μg mL^− 1^ over a 48 h time frame. Progressively with increasing the concentrations of AZG NPs, the 4T1 cells activity decreased gradually. Notably, there was no significant decrease in 4T1 cells activity as the concentrations of AZGH NPs increased. At a concentration of 200 μg mL^− 1^, the cellular viability of cells cultured with AZG NPs was only 80%, whereas the cell survival rate increased by 12% in cultures with AZGH NPs. This observation can be attributed to the enhanced biocompatibility of HA (Fig. 3A) [48, 49]. Subsequently, 4T1 and L929 cells underwent incubation with AZGH NPs across various concentrations and for different durations to further establish their biocompatibility. Remarkably, both cell types exhibited virtually no cytotoxicity (Fig. 3B and C). To further corroborate the nontoxicity of AZGH NPs, flow cytometry was conducted employing Annexin V-FITC/PI staining to cell apoptosis analysis (Fig. 3D). The intracellular endocytosis of AZGH NPs was undertaken as a preliminary step, for the sake of cellular level therapeutic mechanism. Primarily, both AZG NPs and AZGH NPs were labeled with Cy5.5. Subsequently, a series of incubation periods, spanning 1, 4, and 8 h, were employed for treating 4T1 or L929 cells with AZG-Cy5.5 and AZGH-Cy5.5. The resulting fluorescence signal within the cells was then captured using a laser scanning confocal microscope. Compared to the 4T1 cells exposed to AZG-Cy5.5, a robust red fluorescence signal was discerned in those subjected to AZGH-Cy5.5 treatment over the course of time, signifying a heightened uptake of AZGH NPs (Fig. 3E). Furthermore, L929 cells exhibiting diminished CD44 expression demonstrated a notably diminished fluorescence intensity on their cell surfaces compared to the temporal progression observed in 4T1 cells (Figure S10). In order to ascertain that AZGH NPs can produce ROS efficiently, DCFH-DA known as a fluorescent redox probe was utilized to detect ROS in cell experiments. As a cell permeability tracer, DCFH-DA can be oxidized instantaneously upon interaction with ROS to yield extremely high flourescent 2’, 7’-dichlorofluorescein (DCF) molecules [50]. Upon exposure to NIR irradiation, AZ NPs incited a discernible level of ROS production. In comparison, AZG NPs exhibited an augmented ROS generation, as evidenced by the intensified DCF fluorescence, a phenomenon explicable by the synergistic catalytic activity augmentation facilitated by the introduction of GQDs. Notably, the zenith of ROS accumulation was achieved with AZGH NPs, and this heightened ROS production was attributed to the enhanced targeting of 4T1 cells (Fig. 3F). Moreover, intracellular ROS levels were evaluated through flow cytometry analysis, aligning with the heightened ROS levels observed in DCFH-DA analysis (Figure S11). Consequently, the efficacy of AZGH NPs in elevating intracellular ROS levels in 4T1 cells had been established.

### In vitro cellular therapy of the enhanced PDT

**Fig. 4:**
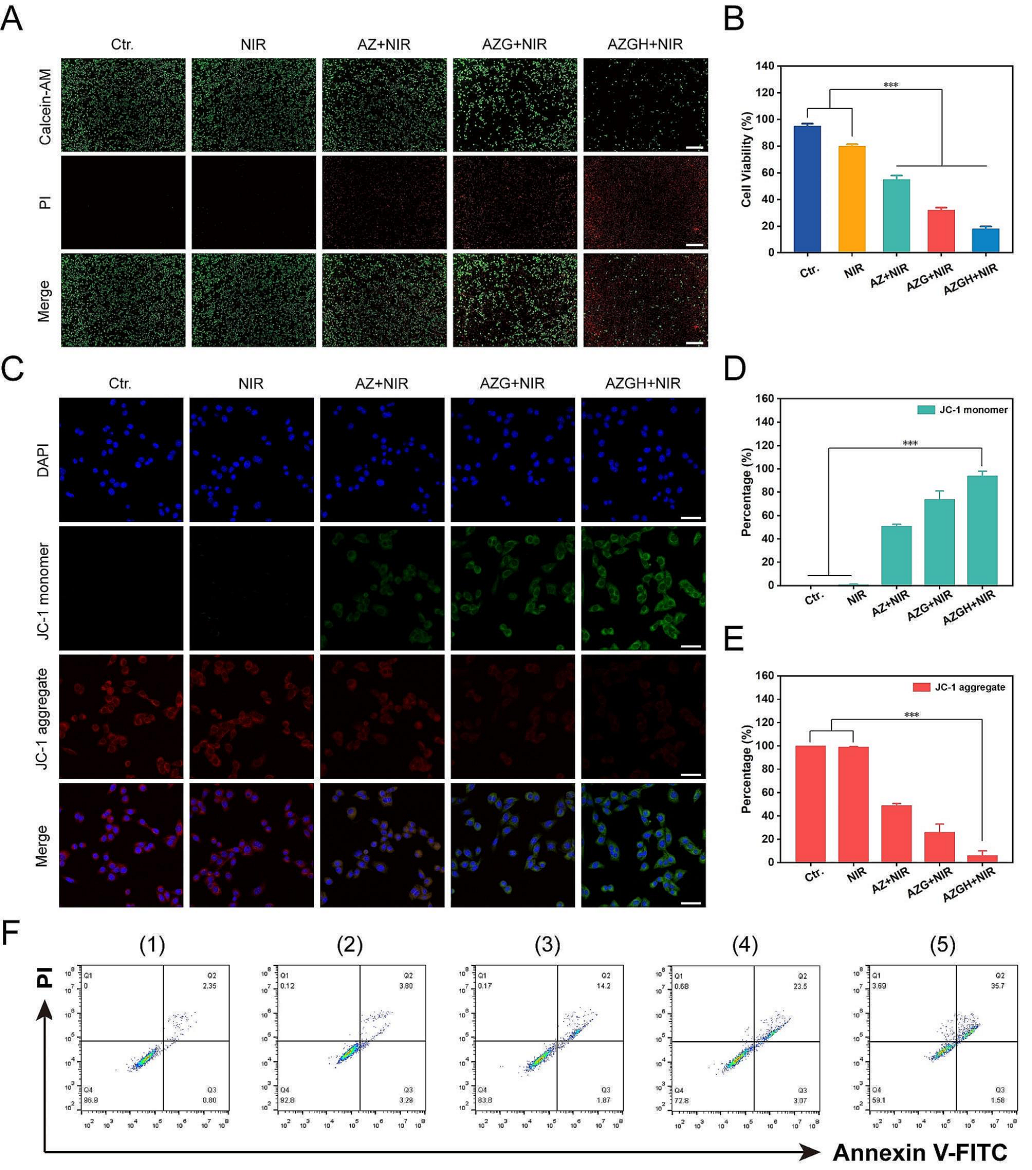
The combined treatment efficacy of AZGH NPs in 4T1 cells. (**A**) 4T1 cells dyed with AM/PI calcein in fluorescence microscope images following different treatments (Scale bar is 100 μm); (**B**) Cell viability of 4T1 cells treated with Control, NIR, AZ, AZG and AZGH NPs. (**C**) The confocal images of 4T1 cells that were treated with JC-1 staining (Scale bar is 50 μm). Fluorescence intensity analysis of monomeric form (**D**) and aggregate form **E**). **F**) The Annexin V-FITC and PI staining assays were performed using flow cytometry after various treatments. (1) control (PBS); (2) NIR; (3) AZ + NIR; (4) AZG + NIR; (5) AZGH + NIR

Afterwards, the investigation of the tumor-specific cytotoxicity induced by AZGH nanoparticles was carried out through a differential fluorescent staining targeting live and deceased cellular populations (Fig. 4A). Within the NIR-treated group, the discernible absence of significant cellular detriment to 4T1 cells was unequivocally established, as substantiated by the prevalent presence of green fluorescent signals within the observational field. In stark contrast, the AZ + NIR group was discerned the sporadic emergence of red fluorescence, meanwhile the AZG + NIR team was indicative of moderate cellular injury discernible. Notably, the AZGH + NIR group exhibited a more pronounced cytotoxic effect. Remarkably, AZGH NPs displayed the most remarkable cellular destruction, attributable to the hybrid plasmonic nano heterostructures that achieved maximum ROS generation. The influence of AZGH NPs was further assessed by a CCK-8 assay. Specifically, AZ, AZG, and AZGH NPs, each at an equivalent concentration, were individually co-incubated with 4T1 cells for 4 h. Subsequently, cells from distinct groups underwent NIR irradiation and were further incubated for 24 h to conduct the CCK-8 experiment for therapeutic evaluation. As depicted in Fig. 4B, pure PBS and NIR irradiation alone exhibited no detrimental effects on the cells, but significant cytotoxicity became evident by the way of the cell subjected to the AZG + NIR group It is reasonable to infer that the combination of AZGH and NIR irradiation manifests the most profound cytotoxicity. In light of the pivotal role of mitochondria in mediating metabolic dysfunction-related cellular injury, an assessment of mitochondrial status was undertaken through the evaluation of mitochondrial membrane potential (MMP) via the utilization of the cationic carbocyanine JC-1 dye [51]. Under the presence of NIR irradiation, AZ NPs induced a modest level of mitochondrial dysfunction, attributed to a limited production of ROS. On the other hand, heightened green fluorescent intensity further confirmed that AZG NPs caused a more substantial degree of mitochondrial damage. Predictably, the most extensive disruption of mitochondrial function was elicited by AZGH NPs, closely aligning with the outcomes of the CCK-8 assay (Fig. 4C). Ultimately, a quantitative evaluation of mitochondrial condition was performed by the calculation of the mean fluorescence intensity (MFI) for both the monomeric form (Fig. 4D) and the aggregate form (Fig. 4E). Moreover, the therapeutic efficacy was also validated through precise flow cytometry apoptosis analysis employing the established Annexin V–FITC/PI dual staining methodology. Notably, the findings underscored that AZGH in conjunction with NIR irradiation exhibited the most pronounced therapeutic outcome (Fig. 4F).

### In vivo biological safety evaluation, targeting effects and phototherapeutic efficacy

**Fig. 5:**
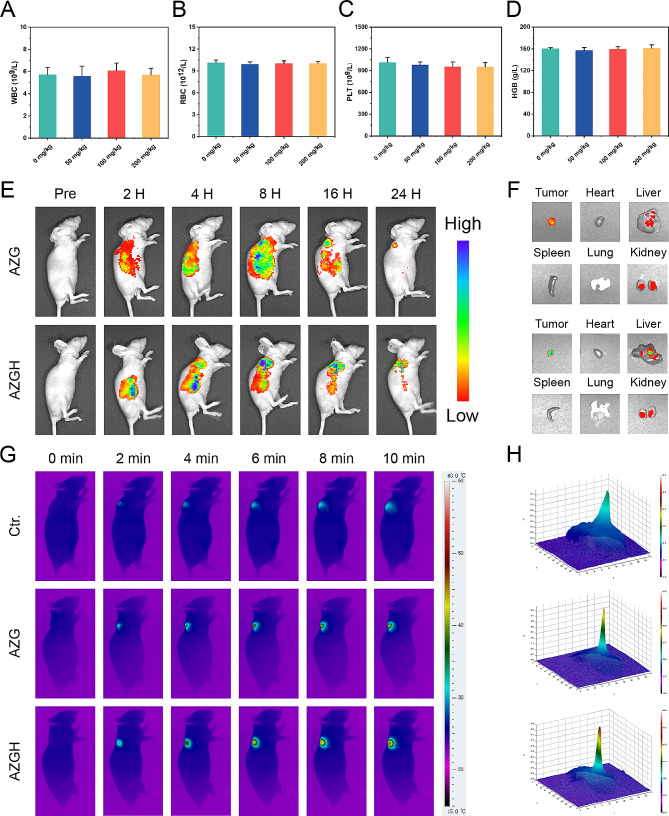
The acute toxicity, targeting effects and phototherapeutic efficacy of AZGH NPs in vivo on Balb/C nude mice. **A**-**D**) Blood routine examination on day 14 following i.v. injection of AZGH NPs at various dosages (0, 50, 100, and 200 mg kg^− 1^). **E**) Real-time fluorescence images captured in 4T1 tumor-bearing mice following intravenous injection of Cy5 labeled AZG NPs and AZGH NPs. **F**) Fluorescence pictures of the tumor and key organs were taken at the middle stage of the examination. **G**) After 24 h of tail vein injection of PBS, AZG or AZGH, the mice were irradiated using a NIR laser for 10 min (NIR: 808 nm, 1.0 W cm^− 2^) and photothermal images within the tumor region were subsequently recorded. **H**) The corresponding 3D photothermal images of the tumor areas of the mice were recorded after 10 min

In anticipation of in vivo investigation into the antitumoral efficacy, a comprehensive assessment of systemic toxicity was executed to validate the biosafety. Initially, a hemolytic assay was employed, wherein AZGH NPs at elevated concentrations of up to 100 mg mL^− 1^ exhibited a low hemolytic rate of less than 5%, thereby affirming their remarkable hemocompatibility (Figure S12). Subsequently, an acute toxicity examination was conducted via intravenous administration of three various dosages of AZGH NPs (50, 100, and 200 mg kg^− 1^) in mice. Upon the 14th day post-intravenous administration, the outcomes stemming from blood biochemistry assays demonstrated unaltered hepatic and renal function indices. Notably, the activities of ALT and AST, alongside BUN and CREA levels, exhibited no discernible deviations in the mice treated with AZGH NPs, as juxtaposed to the control counterpart (Figure S13). Additionally, in the blood routine examination, WBCs, RBCs, HGB, and PLT all remained within the normal range, irrespective of the various doses of AZGH NPs (Fig. 5A-D and S14). In order to facilitate a comprehensive observation of the biological distribution about AZG NPs and AZGH NPs in 4T1 tumor-bearing mice prior to initiation of in vivo therapy, we made use of a fluorescence imaging technique in vivo and ex vivo. As the temporal progression unfolded, both AZG NPs and AZGH NPs displayed a progressive increase in their respective fluorescence intensities. At the 24 h mark post-injection, AZG NPs retained a relatively weak fluorescence, primarily on account of the enhanced permeability and retention (EPR) effect. Conversely, AZGH NPs manifested a heightened fluorescence intensity surpassing that of AZG NPs, signifying a greater accumulation of AZGH NPs within the tumors due to their specific HA-targeting effect (Fig. 5E). In order to gain deeper insights into the metabolism of AZG NPs and AZGH NPs within the mice organs, an examination was conducted on the fluorescence signal present in key organs, including the heart, liver, spleen, lung, kidney, and tumor, 12 h after injection. The presence of fluorescence signal within the tumors suggested a substantial accumulation of AZGH NPs at the tumor sites, thereby augmenting the potential for subsequent therapy (Fig. 5F). Subsequently, in order to more intuitively understand the temperature variations in different nanoparticles after laser irradiation in vivo, we employed infrared thermal imager to capture photothermal image technology for monitoring. The protracted duration of irradiation yielded a discernible augmentation in the PTI signals localized at the tumor site, thereby signifying a substantial elevation in temperature. After 10 min laser irradiation, a rapid elevation in temperature was distinctly observed in the tumor site of the murine model, reaching ∼ 49 °C in the AZG NPs and ∼ 44 °C with AZGH NPs (Fig. 5G and S15). Notably, the cellular membrane of 4T1 cells was adorned by an abundance of CD44 receptors, thereby elucidating the conspicuous thermal disparity discernible at the tumor locus across the two aforementioned NPs. This receptor-rich condition substantially expedited the intracellular uptake of AZGH NPs, resulting in a substantial enhancement of active targeting. Distinct 3D photothermal images were obtained for each group, depicting temperature fluctuations (Fig. 5H). In conclusion, the excellence of PT capacity in AZGH NPs was unequivocally validated.

### Antitumor effect in vivo

**Fig. 6:**
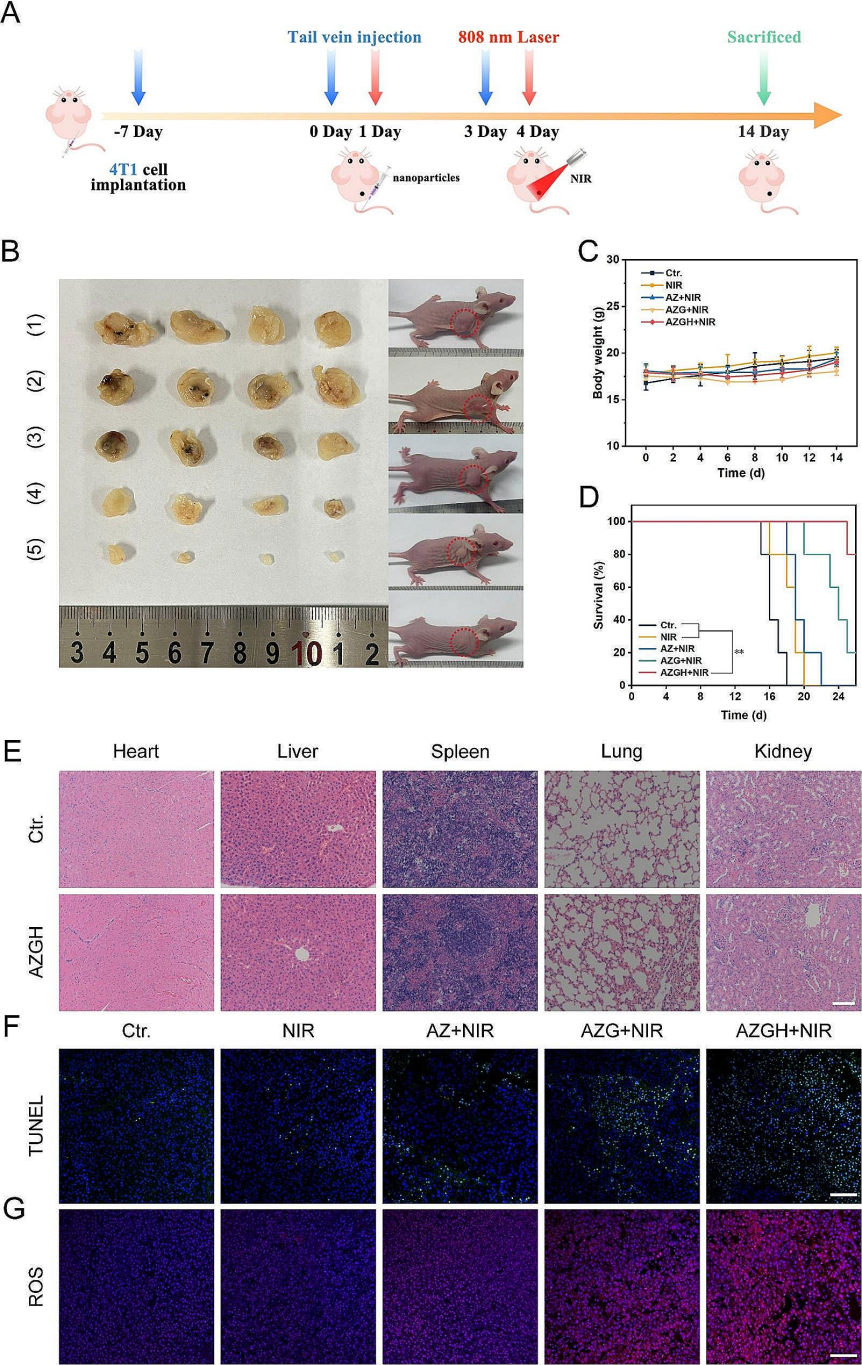
In vivo antitumor efficacy in 4T1 tumor-bearing mice. (**A**) Schematic illustration and timetable for the cancer treatment. (**B**) Photographs of the mice and tumors following the various groups. (1) control (PBS); (2) NIR; (3) AZ + NIR; (4) AZG + NIR; (5) AZGH + NIR. Body weight curves **C**), and curves of survival **D**) in mice given therapy. **E**) H&E staining of the key organs slices in Ctr. and AZGH groups (Scale bar is 100 μm). **F**) TUNEL staining of the tumor slices in different groups (Scale bar is 100 μm). **G**) ROS staining of the tumor slices in different groups (Scale bar is 100 μm)

Motivated by the remarkable cytotoxicity exhibited at the cell-based assays, the therapeutic effectiveness of AZGH NPs in inhibiting tumor growth was exhaustively evaluated in murine model harboring 4T1 tumor. To this end, the murine model was initially injected with AZGH NPs (equivalent AZ and AZG content) through the tail vein, followed by NIR irradiation directed towards the lesion site 24 h post injection (Fig. 6A). In comparison to the PBS control group, it became evident that NIR irradiation had little impact on altering tumor growth, signifying a marginal impact of exogenous stimulation throughout the treatment regimen. Conversely, the expansion of tumors was partly impeded through the application of AZ + NIR and AZG + NIR, owing to the manifestation of their cytotoxic PDT effects. Remarkably, the AZGH + NIR group showed the most fascinating suppression impact on tumor growth after a span of 14 days post inoculation, displaying a remarkably advanced tumor inhibition rate of 94.2%, a phenomenon likely ascribed to the enhanced ROS generation induce 4T1 cells apoptosis (Fig. 6B). Furthermore, our findings demonstrated that the survival rates of mice subjected to AZGH NPs injection surpassed 75% upon exposure to NIR irradiation. However, all mice treated with NIR only died after 20 days, which indicated the remarkable anti-tumor efficiency of AZGH NPs under NIR irradiation (Fig. 6D). Upon the conclusion of the therapeutic intervention, the potent eradication of tumor by AZGH NPs was additionally corroborated through the quantitative assessment of solid tumors from dissected mice (Fig. 6B and S16). Concurrently, the mice body weight exhibited no significant fluctuations throughout the treatment duration, indicating a negligible deleterious impact of said therapeutic regimen on murine metabolic homeostasis (Fig. 6C). Histological examination employing hematoxylin and eosin (H&E) stained sections of vital organs, including the heart, liver, spleen, lung, and kidney derived from the entire cohort of mice, revealed an absence of any discernible pathological anomalies, thereby underscoring the remarkable biocompatibility of AZ, AZG, and AZGH NPs (Fig. 6E and S17). Finally, to elucidate the in vivo tumor inhibition mechanism of the AZGH nanase combined with NIR irradiation strategy, we conducted TUNEL and ROS staining on diverse sets of tissue sections (Fig. 6F and G). Employing the TUNEL technique to visualize processed DNA fragments, we observed that AZGH nanase, when coupled with NIR irradiation, induced apoptosis and denucleation in the majority of tumor cells, underscoring the substantial DNA damage inflicted (Fig. 6F). DHE fluorescence demonstrated a noteworthy increase in ROS levels within tumor tissues upon simultaneous exposure to AZGH NCs and NIR (Fig. 6G). These findings collectively suggest that AZGH NRs exert a tumor growth inhibitory effect on breast cancer without causing significant harm to vital organs during treatment.

## Conclusions

In brief, to demonstrate the feasibility and effectiveness of synchronous PDT and PTT treatments for TNBC, we synthesized AZGH NRs possessing good biocompatibility. The nano heterostructures of AZGH is established through the integration of GQDs into AZ core-shell structure and successfully exploited in advancing tumor elimination via a ROS generation mechanism. Comparative analysis with AZ NPs reveals that the as-synthesized AZG NPs heterostructure enhances ROS production when subjected to NIR actuation. This augmented efficiency in generating ^1^O_2_ and •OH is attributed to the rapid separation of e^−^-h^+^ pairs. Moreover, the decorated HA can target 4T1 cells and increase the content of AZGH decomposition in 4T1 cells, and the photocatalytic activity effectively play the role in combination therapy. Meanwhile, Au NRs, acknowledged as exemplary PTT agents, demonstrate pronounced light absorption within the NIR spectrum, efficiently instigating apoptosis in tumor cells. The results have demonstrated that the designed AZGH NPs has potent ability to eradicate tumor subjected to NIR irradiation. This study introduces a promising approach to design an efficient photosensitizer for enhancing photocatalysis therapy through the facilitation of electrons transfer for clinical application.

### Electronic supplementary material

Below is the link to the electronic supplementary material.

Supplementary Material 1: Materials, Apparatus, Synthesis of Au NRs, Characterization of AuNR@ZnO@GQDs-HA, Photothermal performance in vitro, Toxicity and safety studies in vitro, Mitochondrial integrity assay

## Data Availability

No datasets were generated or analysed during the current study.
