# Supplementary material for: Ternary heterostructure-driven photoinduced electron-hole separation enhanced oxidative stress for triple-negative breast cancer therapy
Source: J Nanobiotechnology. 2024 May 12;22:240. doi: 10.1186/s12951-024-02530-4 (PMC11089806; doi:10.1186/s12951-024-02530-4)
Supplement: Supplementary file 1 — Supplementary Material 1: Materials, Apparatus, Synthesis of Au NRs, Characterization of AuNR@ZnO@GQDs-HA, Photothermal performance in vitro, Toxicity and safety studies in vitro, Mitochondrial integrity assay [file 12951_2024_2530_MOESM1_ESM.doc]

**Supporting Information**

Ternary Heterostructure-Driven Photoinduced Electron-Hole Separation Enhanced Oxidative Stress for Triple-Negative Breast Cancer Therapy

Shuqing Dong^1,2,3^, Yuqi Huang^2^, Hanrong Yan^2^, Huarong Tan^2^, Liying Fan^2,3^, Minghao Chao^2^, Yiping Ren^2^, Ming Guan^1^,*, Jiaxin Zhang^3^,*, Zhao Liu^3^,* and Fenglei Gao^2^,*

1. Department of Laboratory Medicine, Huashan Hospital, Shanghai Medical College, Fudan University, Shanghai 200040, China.

2. Jiangsu Key Laboratory of New Drug Research and Clinical Pharmacy, Xuzhou Medical University, 221004, Xuzhou, China

3. Department of Thyroid and Breast Surgery, Affiliated Hospital of Xuzhou Medical University, 221004, Xuzhou, China

Email: [guanming88@yahoo.com](mailto:guanming88@yahoo.com) (M. Guan), jsxzgfl@sina.com (F. Gao), xylzhao9999@163.com (Z. Liu), zhangjiaxin1969@163.com (J. Zhang).

**Contents**

**Materials**……………………..………….………………………………….......S4

**Apparatus**………………………………………………………………………S4

**Synthesis of Au NRs**………..…..…..…...…..……………………..…..….……S5

**Characterization of AuNR@ZnO@GQDs-HA** ……….………………….… S5

**TMB evaluates the generation of •OH with AZGH** .….………………….… S6

**Photothermal performance in vitro** ……..…..…..…..……..…………….…..S6

**Toxicity and safety studies in vitro**…………………………….…….....….….S7

**Mitochondrial integrity assay** .....….………………………………….....……S7

**TEM Images (S1)**.. ..…………………………………………………………...S9

**Luminescence Properties of QDs (S2)**……….………….…….....….…… ..…S9

**Diameters of AZGH NPs (S3)**……….………….…….....….……………....…S10

**High resolution XPS spectra (S4)** ……….………….…….....….………..….. S10

**EDS of AZGH NPs (S5)**………………………………………………………..S11

**FT-IR spectra (S6)** ...…………………………………………………………...S11

**Steady-state PL spectra for AZ and AZG NPs (S7)** …….……….….……… S11

**•OH generation under different AZGH NPs concentrations (S8)** ….……... S12

**NIR thermal images of AZGH NPs (S9)**……………………………………...S12

**Fluorescence images of L929 cells (S10)** ……………………………………..S13

**Flow cytometry analysis ROS production (S11)** …………………………….S13

**Hemolysis (S12)**...………………………………………………………….…...S13

**Blood biochemistry (S13)**.. ……………………………………………….…...S14

**Blood routine examination (S14)** …………………………………………….S14

**Photothermal curves of tumor (S15)** …….………………………………….S14

**Tumor weight and volume (S16)**……….………………………………….…S15

**H&E Staining (S17)** …………………………………………….……………S15

**Materials**. Cetyltrimethyl ammonium bromide (CTAB), ascorbic acid (AA), gold (III) chloride trihydrate (HAuCl_4_·3H_2_O), silver nitrate (AgNO_3_), sodium borohydride (NaBH_4_, 99%), zinc nitrate (Zn(NO_3_)_2_•6H_2_O), HMT were purchased from Aladdin. RPMI 1640, 0.25% trypsin-EDTA and paraformaldehyde solution were obtained from Vicmed. Fetal bovine serum (FBS) was obtained from Gibco. Calcein-AM and propidium iodide (PI), 2,7-dichlorofluorescein diacetate (DCFH-DA), CCK-8 reagent, 4′,6-diamidino-2-phenylindole (DAPI), JC-1 staining kit, and annexin V-FITC/PI apoptosis detection kit were purchased from KeyGEN BioTECH. Singlet oxygen sensor green (SOSG) was brought from Sigma-Aldrich. All the other chemicals and reagents were analytical grade and used as received. Deionized water of ~18.2 MΩ·cm was used for all the experiments.

**Apparatus.** Transmission electron microscope (TEM) was carried out using a FEI Tecnai T20 transmission electron microscope. X-ray photoelectron spectroscopy (XPS) measurement was carried out using an ESCALAB 250 instrument to analyze the valence of the Au, Zn, O and C component of AZGH NPs. Malvern Zetasizer Nano ZS90 was used to measure the zeta potential and dynamic light scattering of various samples. Energy dispersive X-ray spectroscopy (EDX) and element mapping were tested by Zhong Ke Bai Ce. UV-Vis-NIR absorption spectra was performed on UV-1601 spectrophotometer. Bio-Rad 680 microplate reader was utilized to standard CCK-8 assay. Trapping of the generated •OH and ^1^O_2_ at room temperature by an electron spin resonator (ESR, Magnettech MS5000, Bruker, Germany). Fluorescence inverted microscope (IX73P1F, Olympus, Japan) and confocal laser scanning microscope (CLSM, Leica STELLARIS 5, Germany) were employed for observation. Flow cytometric analysis was performed on a BD FACSAria™ Fusion. An IVIS imaging system (Lumina S5, USA) was adopted for living body fluorescence imaging.

**Synthesis of Au NRs.** A seed-mediated strategy was used to synthesize Au NRs. (Nikoobakht and El-Sayed, 2003) The synthesis process commenced with the swift injection of 0.6 mL of 0.01 M ice-cold NaBH4 solution freshly prepared was carried out into a mixture comprising 250 μL HAuCl4 (0.01 M) and 10 mL CTAB (0.1 M). This step yielded a seed solution with a characteristic brownish-yellow hue. Subsequently, the seed solution was allowed to stabilize at ambient temperature for a minimum duration of 2 h. In the subsequent phase, 2.0 mL HAuCl4 (0.01 M), 0.38 mL AgNO3 (0.01 M), 0.8 mL HCl (1.0 M), 0.32 mL ascorbic acid (0.1 M), and 96 μL of the aforementioned seed solution were meticulously added into a solution comprising 40 mL CTAB (0.1 M). Following this addition, a period of uninterrupted longitudinal growth was allowed for no less than 6 hours. This meticulous procedure culminated in the successful production of CTAB-capped Au NRs.

**Characterization of AuNR@ZnO@GQDs-HA.** A transmission electron microscope of the FEI Tecnai T20 model was employed to achieve the morphology's TEM images. Meanwhile, the scanning electron microscopy (SEM) images was facilitated through the utilization of a field-emission electron gun integrated into the Hitachi S4800 SEM instrument. To probe the valence states of constituent elements, specifically Zn, Au, O, and C within the AuNR@ZnO@GQDs NPs, X-ray photoelectron spectroscopy (XPS) spectra were meticulously acquired employing the ESCALAB 250 spectrometer. Furthermore, determination of particle size characteristics was accomplished through the application of a Malvern Zetasizer Nano ZS90 analyzer. The UV–vis absorbance spectra was obtained by means of a UV-1601 spectrophotometer. Bio-Rad 680 microplate reader was utilized to standard CCK-8 assay. The photothermal efficacy of both the nanoparticles and mice was assessed utilizing an infrared thermal camera (FLIR System E40). For in vivo fluorescence imaging of living body, an IVIS imaging system (Lumina S5, USA) was employed. The capture and detection of the resultant •OH and 1O2 at ambient temperature were accomplished by an electron spin resonator (ESR, Magnettech MS5000, Bruker, Germany). Microscopic observations were conducted using a fluorescence inverted microscope (IX73P1F, Olympus, Japan) and a confocal laser scanning microscope (CLSM, Leica STELLARIS 5, Germany).

**TMB evaluates the generation of •OH with different concentrations of AZGH.** To elucidate the generation of •OH species facilitated by AZGH NPs, TMB (0.5 × 10^−3^ M) served as the •OH indicator. The UV–vis–NIR spectra of the specimens were systematically surveyed to track the absorbance of TMB at 652 nm, serving as a discernible measure of •OH production. Following a 30-second interval, a subsequent examination of the UV–vis–NIR spectra allowed for the scrutiny of TMB's absorbance at 652 nm, signifying the onset of •OH generation.

**Photothermal performance in vitro.** Utilizing 1 mL AZGH nanoparticles, prepared at varying concentrations within an aqueous solution, we conducted an assessment of the photothermal attributes of these nanoparticles. Employing a laser operating at a wavelength of 808 nm, the samples underwent irradiation over a 10-minute interval, with power densities spanning from 0.25 to 1.25 W cm⁻². Concurrently, we monitored the temperature fluctuations in the dispersed AZGH solution at 30-second intervals, employing infrared thermography (Fotric). Subsequently, AZGH NPs (100 μg mL⁻¹) were exposed to 808 nm laser radiation for a duration of 10 minutes. Upon cessation of laser irradiation, the suspension was allowed to gradually equilibrate to room temperature, and this procedure was iteratively replicated to assess the stability of photothermal. Every trial was performed in triplicate.

**Toxicity and safety studies *in vitro*.** 4T1, one of the breast carcinoma cell lines, and L929, one of the fibroblast cell lines, were harnessed to evaluate cellular viability. These cells, which known as L929 and 4T1, were separately cultivated in DMEM (10% FBS added) and Roswell Park Memorial Institute 1640 (RPMI-1640) (10% FBS added), under constant conditions of 37°C and an atmosphere with 5% CO2. The AZGH NPs cytotoxicity in vitro was assessed utilizing a conventional CCK-8 assay. Specifically, both cells were plated in 96-well microplates, with each well containing either 1×10^4^ cells, and left incubate overnight. After, 4T1 and L929 cells were treated with AZ, AZG, or AZGH (12.5, 25, 50, 75, 100, 200 μg mL−1) for 24 h. Cells were incubated for an extra 2 hours after the culture medium had been exchanged to CCK-8 reagent (10 µL). A Bio-Rad 680 microplate was employed to detect the absorbance of each well.

**Mitochondrial integrity assay.** The 4T1 cells were plated on Petri dishes with a density of 50000 cells per for overnight incubation. Subsequently, the cells were divided into 5 groups, categorized as follows: (1) control (PBS), (2) NIR, (3) AZ+NIR, (4) AZG+NIR, and (5) AZGH+NIR. Cells were incubated for an extra 1 hour after the culture medium had been exchanged to JC-1 staining reagent. Then, DAPI was used to stain the cellular nuclei about 5 minutes. Ultimately, Fluorescence images of the cells can be captured and imaged utilizing CLSM.

**
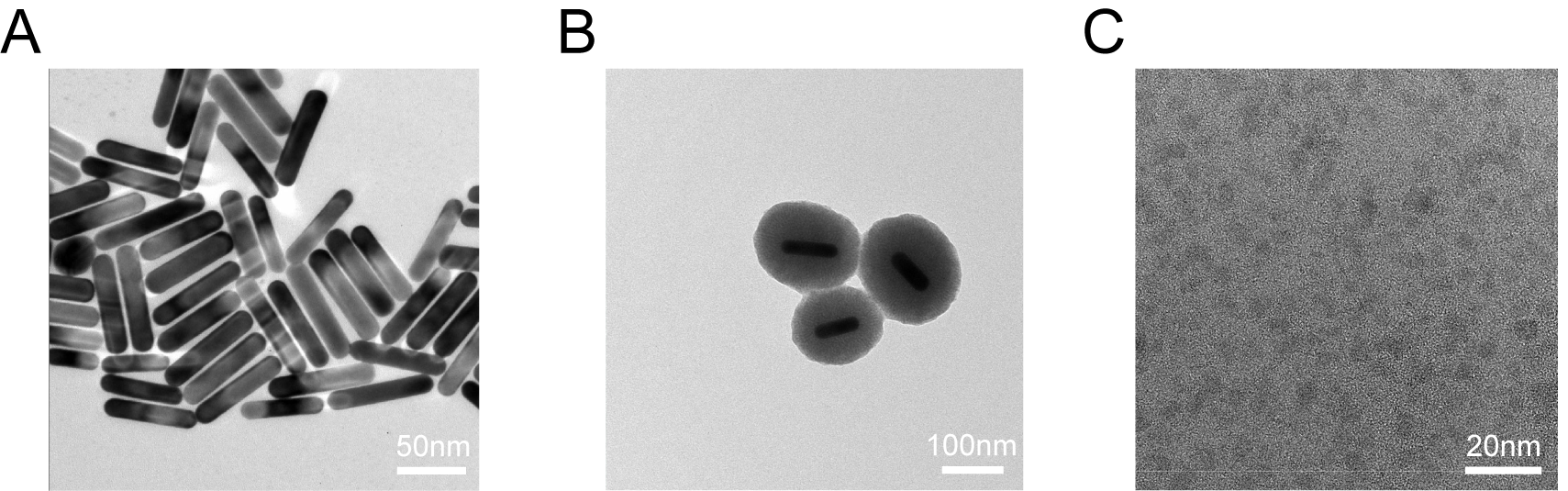
**

**Figure S1** A) TEM image of Au NPs, scale bar is 50 nm. B) TEM image of AZ NPs, scale bar is 100 nm. C) HRTEM image of GQDs NPs, scale bar is 20 nm.


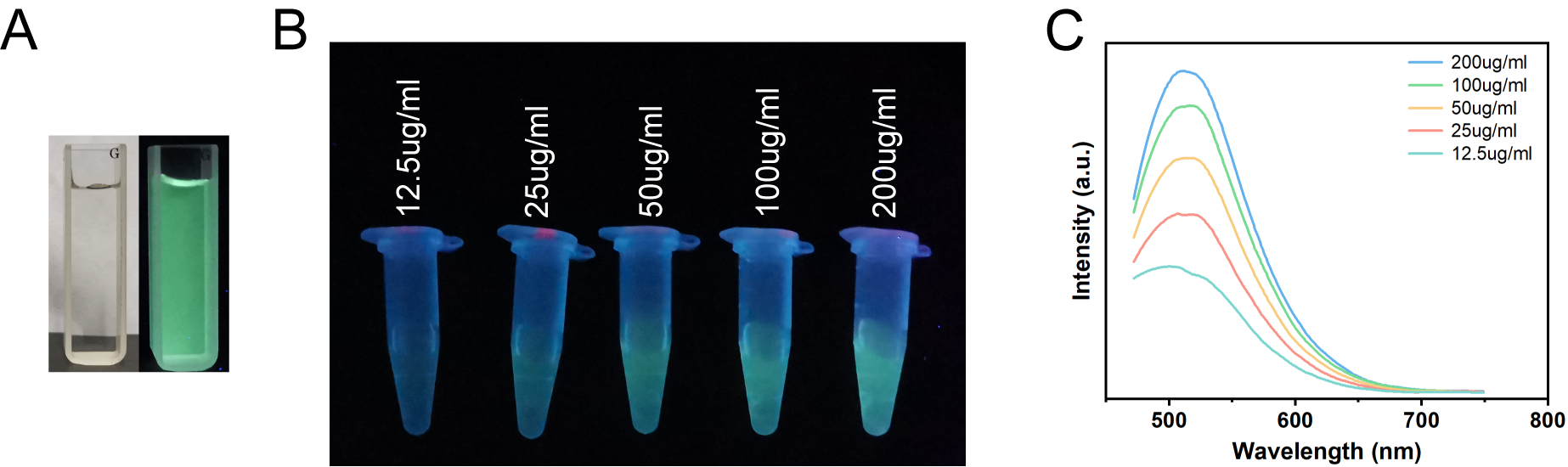


**Figure S2** A) Photograph of the GQD aqueous solution taken under UV light in a fluorescence spectrophotometer. B) Photograph and C) composition-tunable PL spectra of GQDs by changing the concentration. The concentration was 12.5, 25, 50, 100 and 200 μg/mL, respectively.


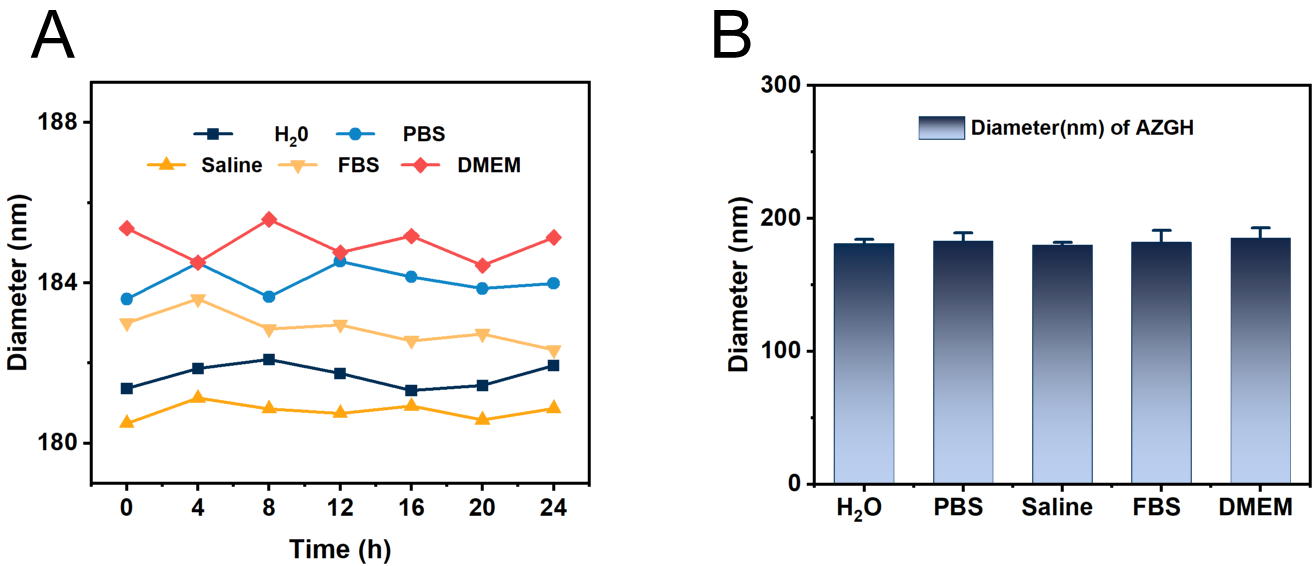


**Figure S3** Diameters of AZGH NPs following a 24 h period of stand in PBS, Saline, FBS and RPMI 1640 solutions.


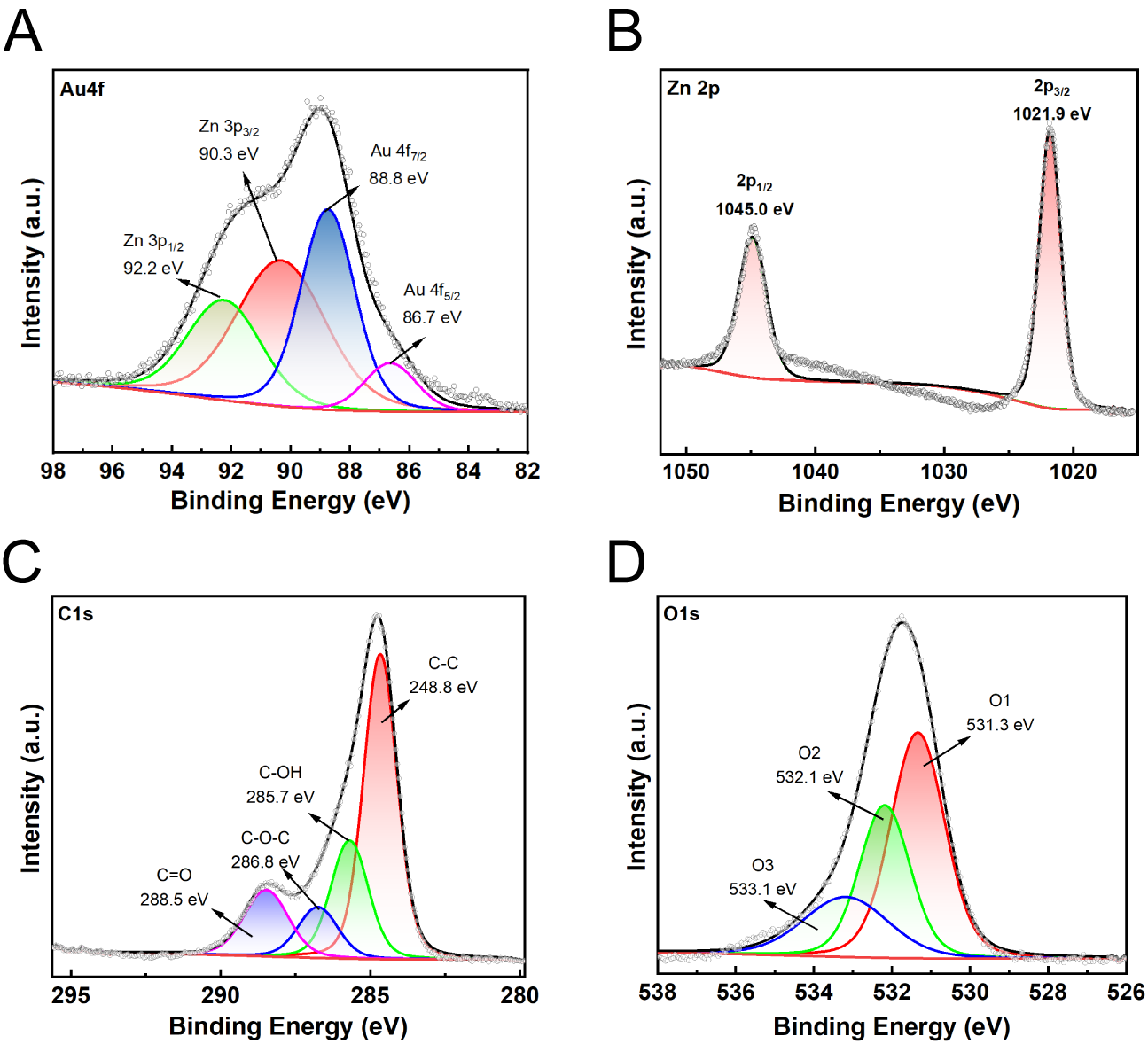


**Figure S4** High resolution XPS spectra of A) Au 4f, B) Zn 2p, C) C 1s, D) O 1s.


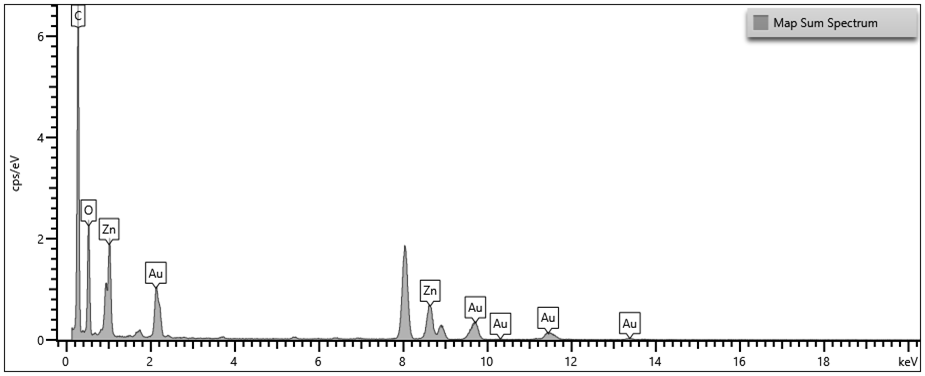


**Figure S5** EDS of AZGH NPs.


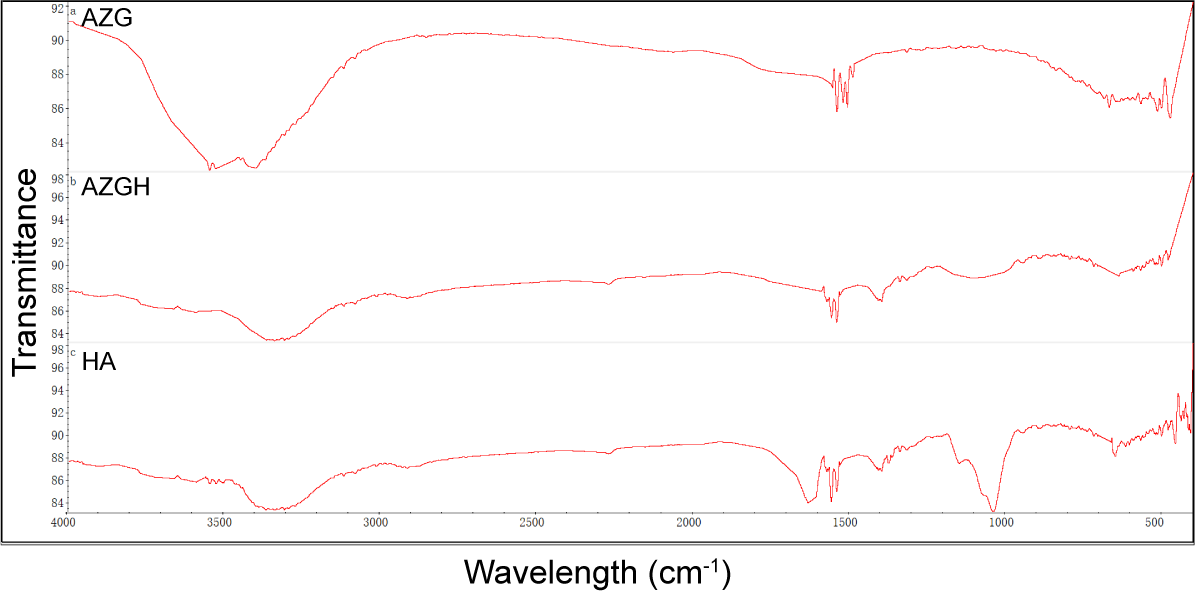


**Figure S6** FT-IR spectra of HA, AZG, and AZGH, respectively.


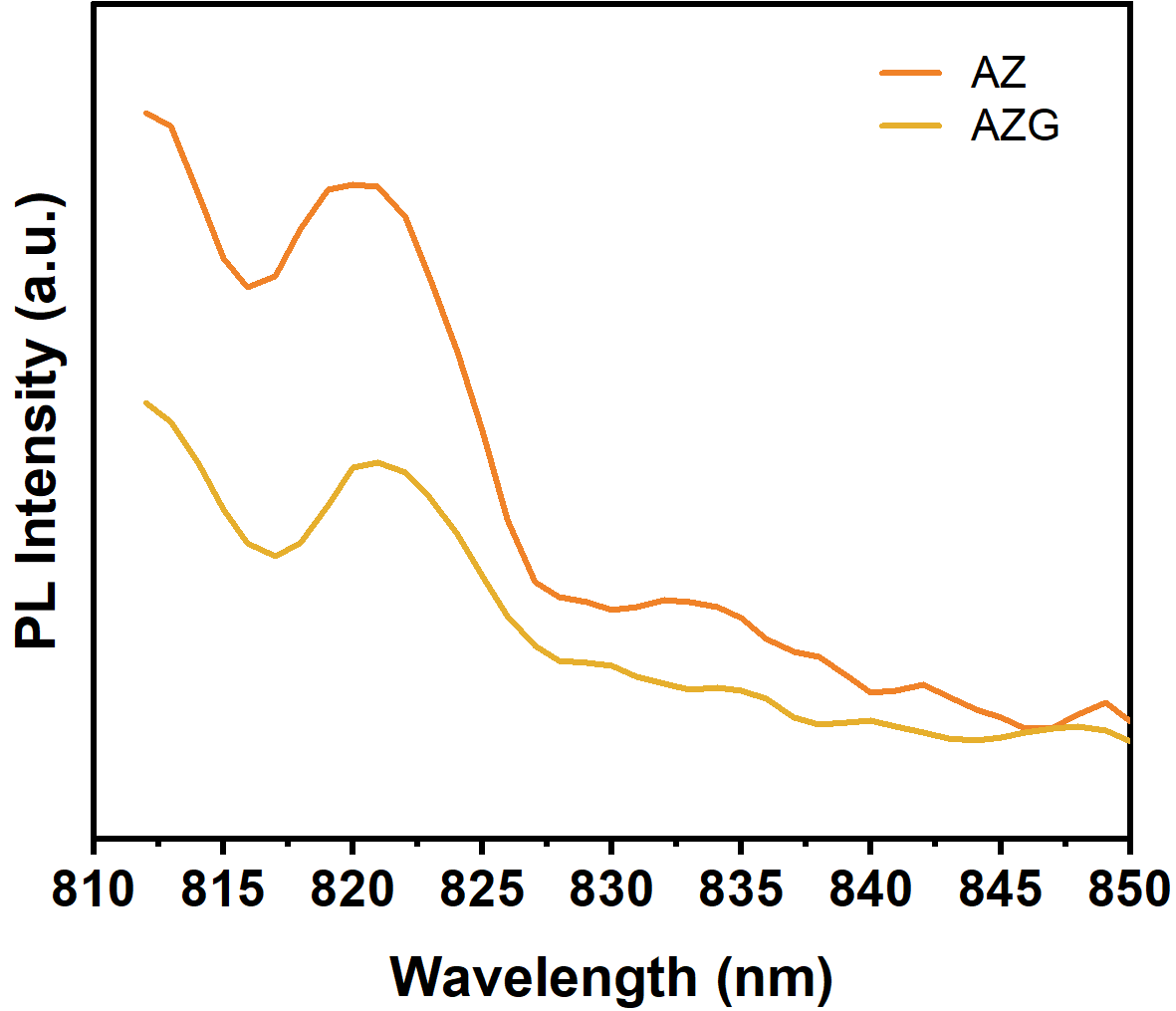


**Figure S7.** Steady-state PL spectra for AZ and AZG NPs.


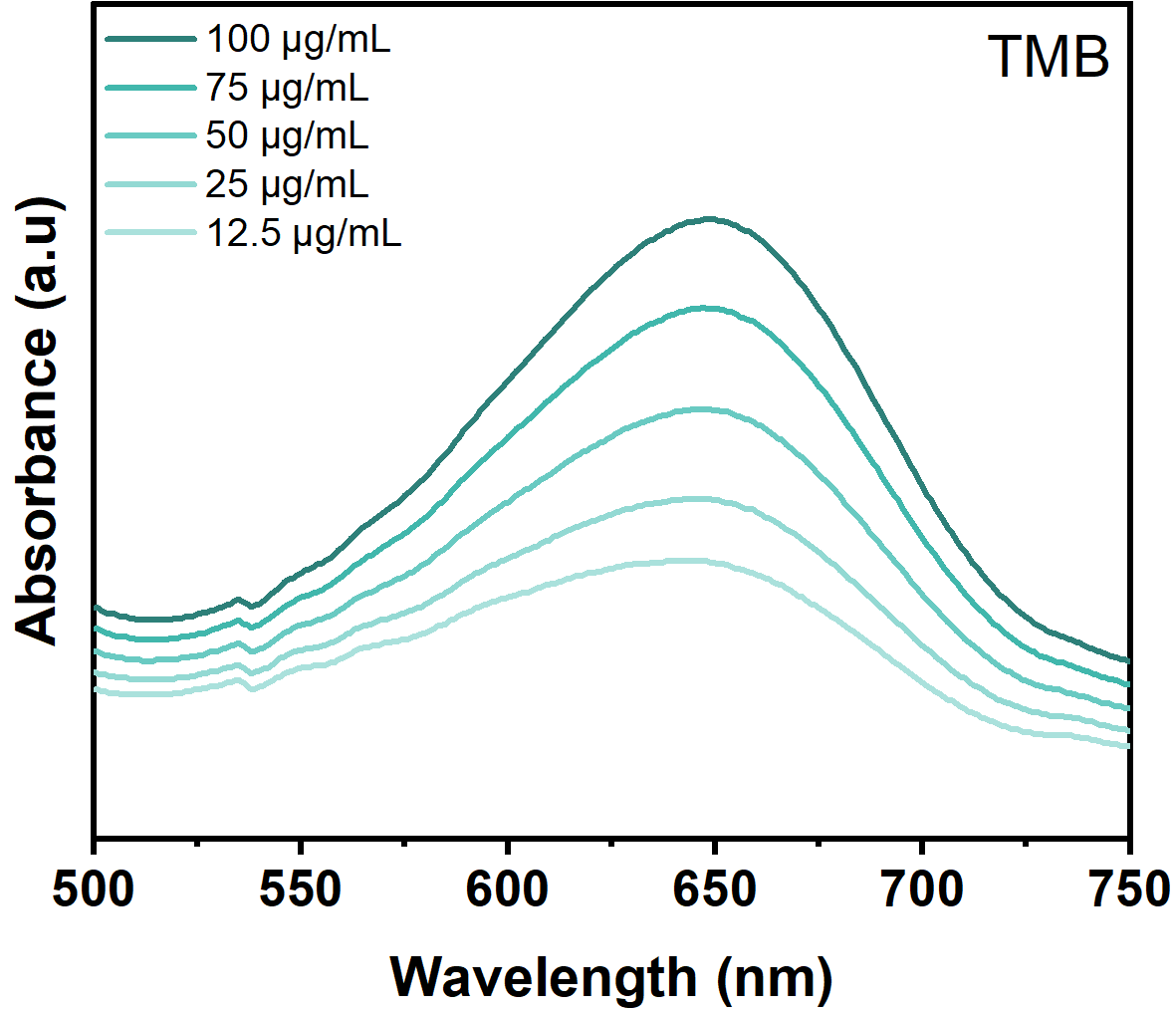


**Figure S8.** Production of •OH under different AZGH NPs concentrations using TMB as the probe.


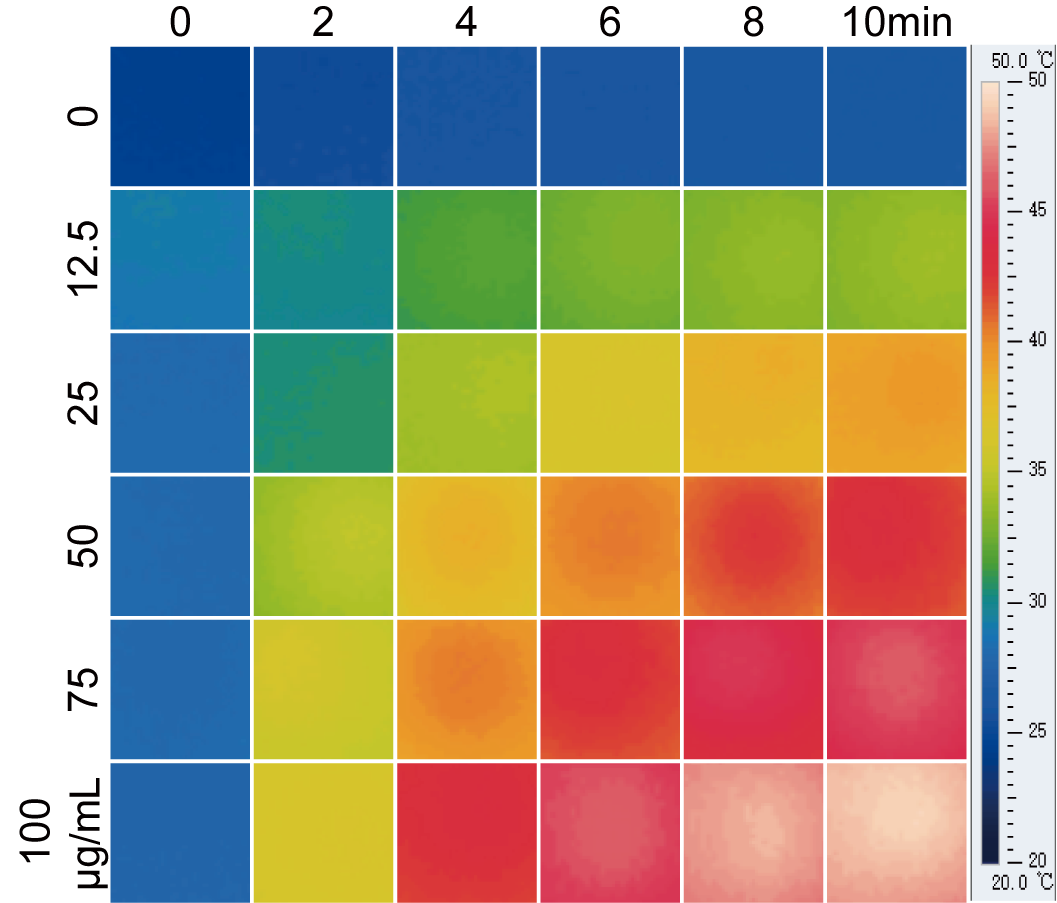


**Figure S9** NIR thermal images of AZGH NPs aqueous solutions at disparate concentrations (0, 12.5, 25, 50, 75 and 100 μg/mL) under the NIR laser irradiation (808 nm, 1.0 W cm^− 2^) for 10 min.


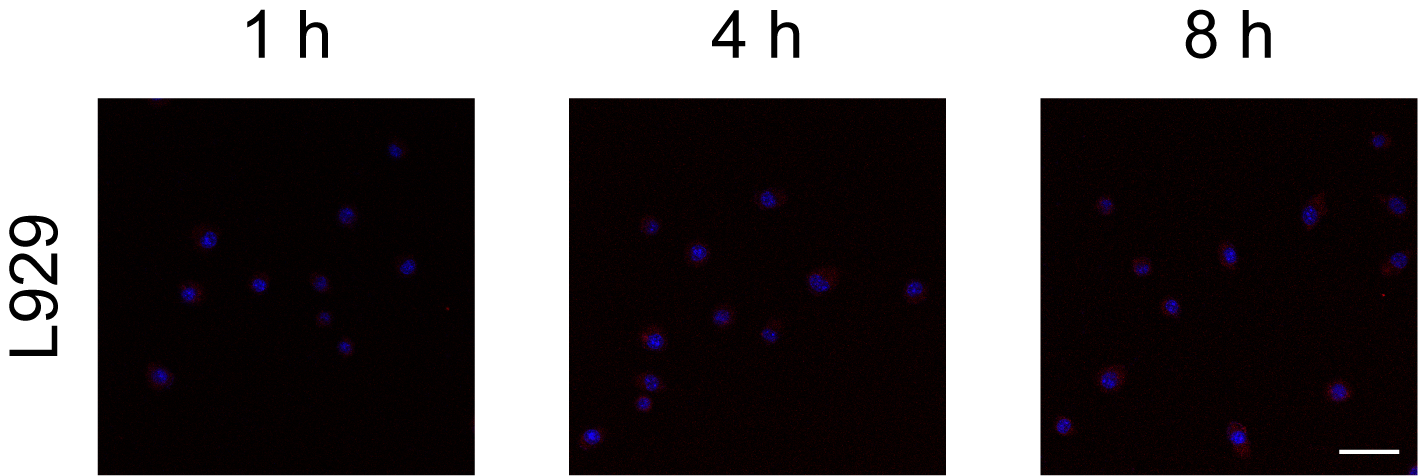


**Figure S10.** Fluorescence images of L929 cells co-incubation with AZGH NPs after 1, 4 and 8 h intervention (Scale bar is 50 µm).


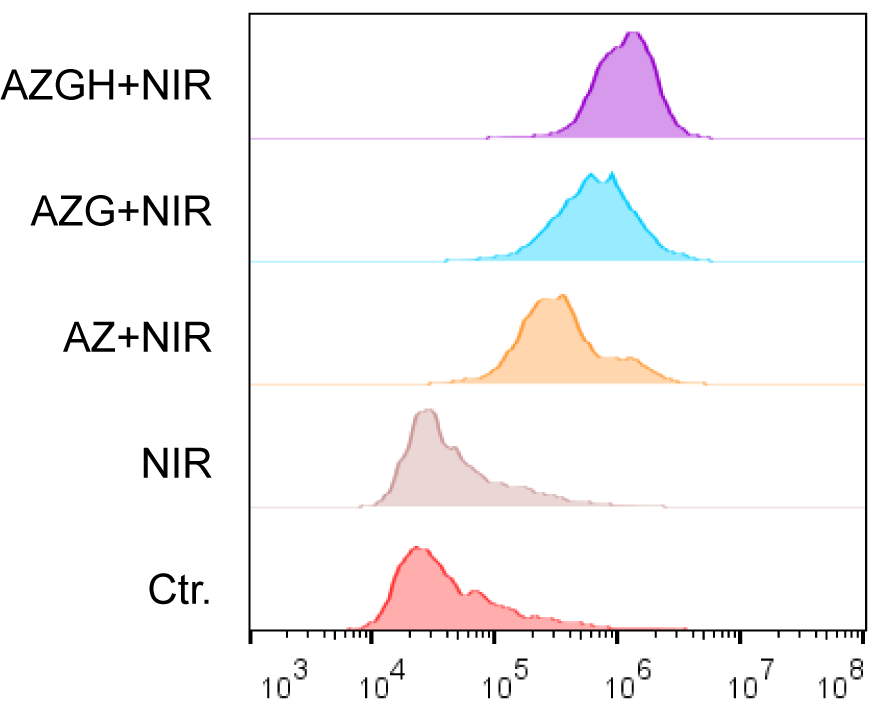


**Figure S11.** Flow cytometry analysis ROS production in 4T1 cells stained with DCFH-DA.


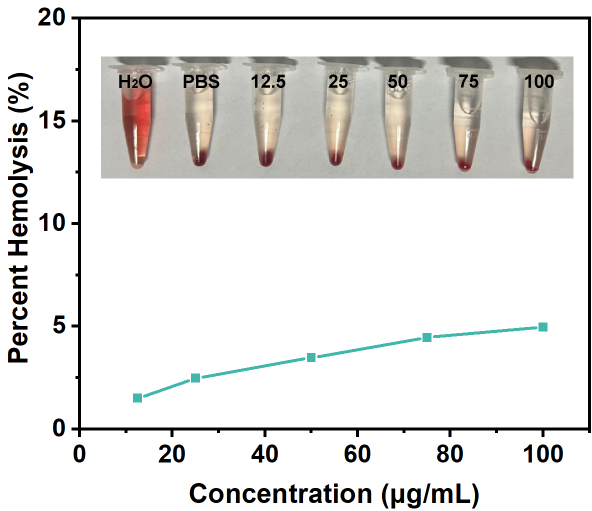


**Figure S12** Hemolysis and absorbance following the introduction of varying concentrations of AZGH NPs.


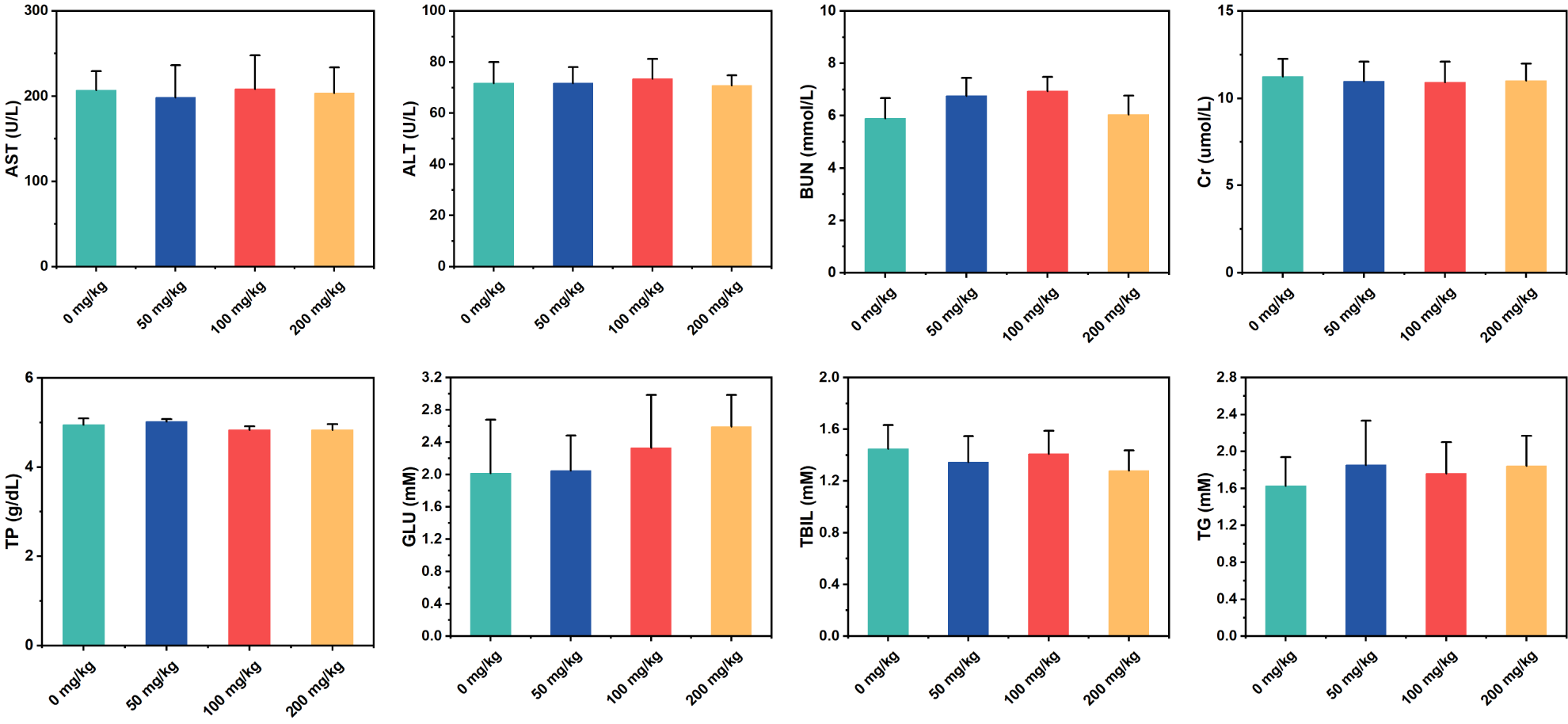


**Figure S13** Blood biochemistry analysis after injection of AZGH NPs with different concentrations.


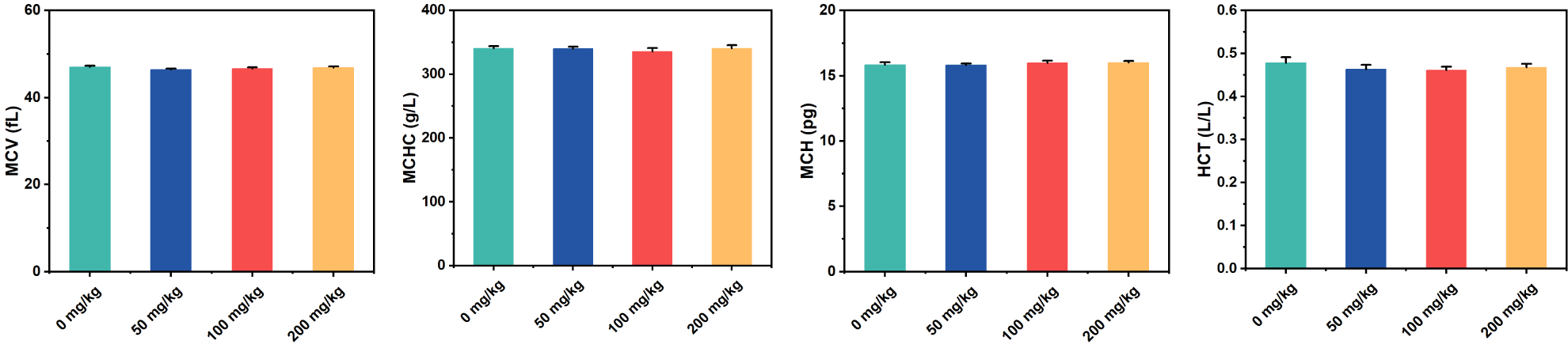


**Figure S14.** Blood routine examination on day 14 following i.v. injection of AZGH NPs at various dosages.


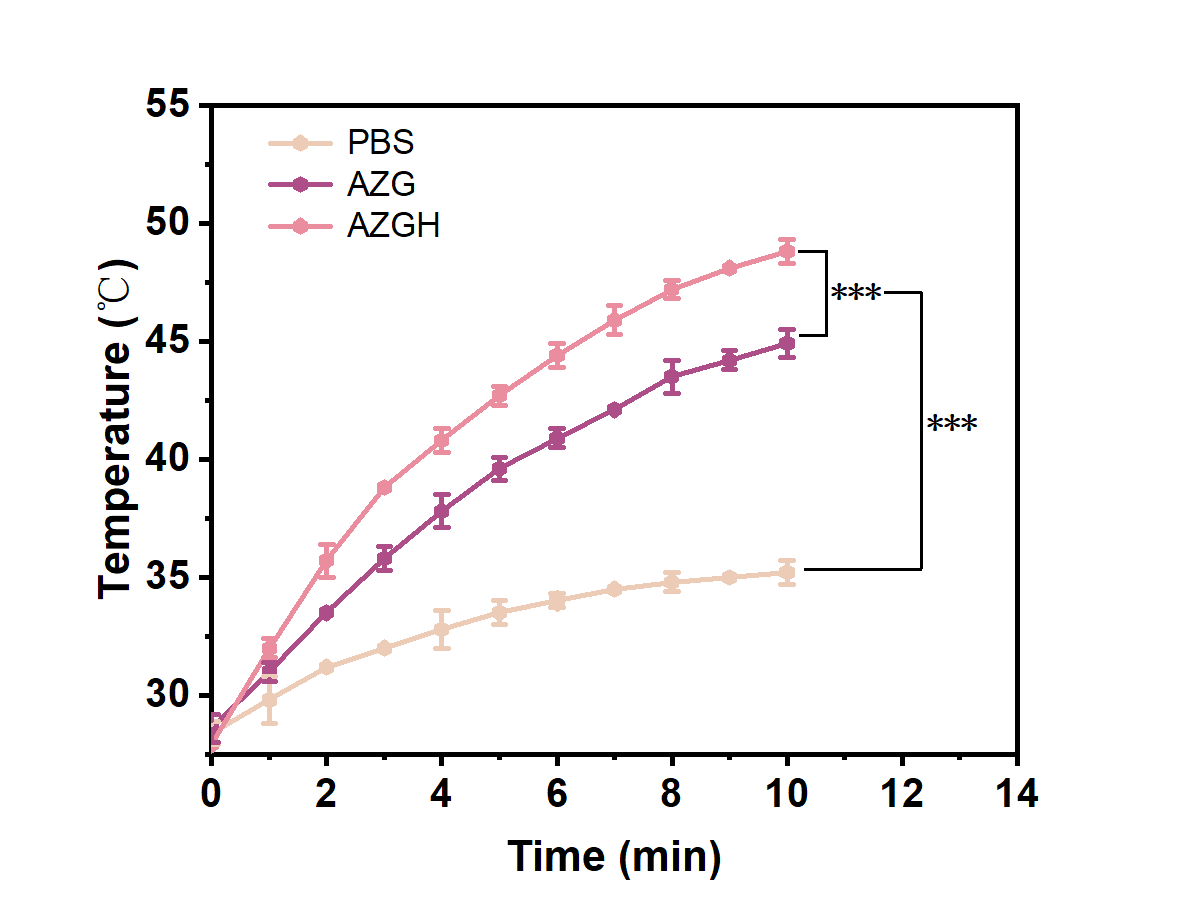


**Figure S15.** Photothermal curves of tumor bearing nude mice after different treatments.


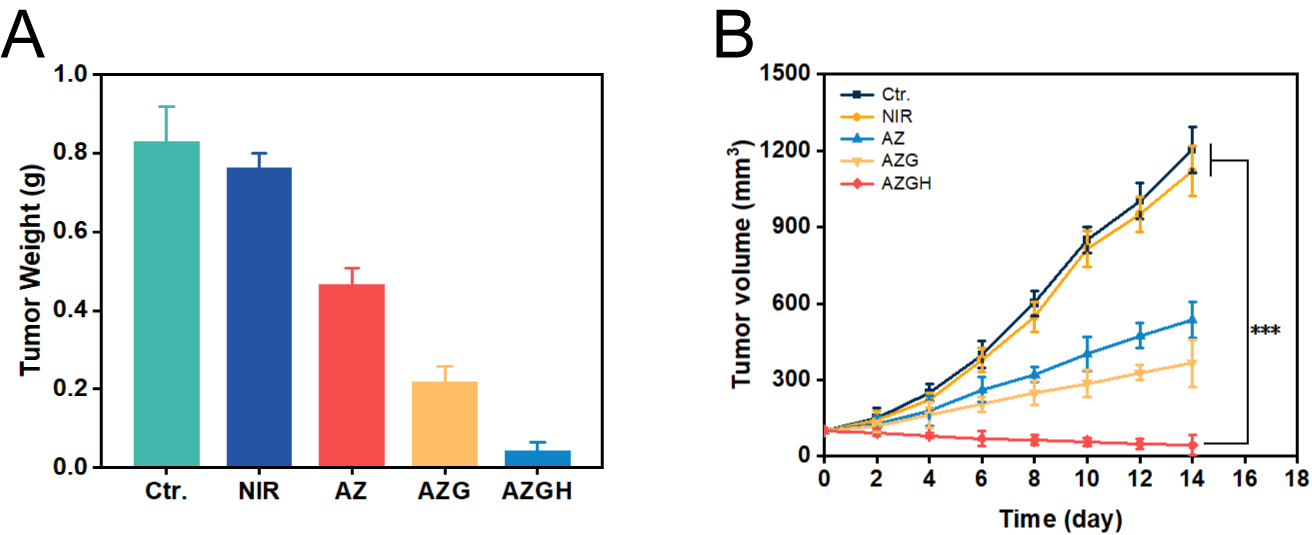


**Figure S16** A) Tumor weight and B) tumor volume of 4T-1 tumor-bearing mice over 14 days assessment interval with diverse therapeutic modalities. Data are mean ± SD (n = 4).


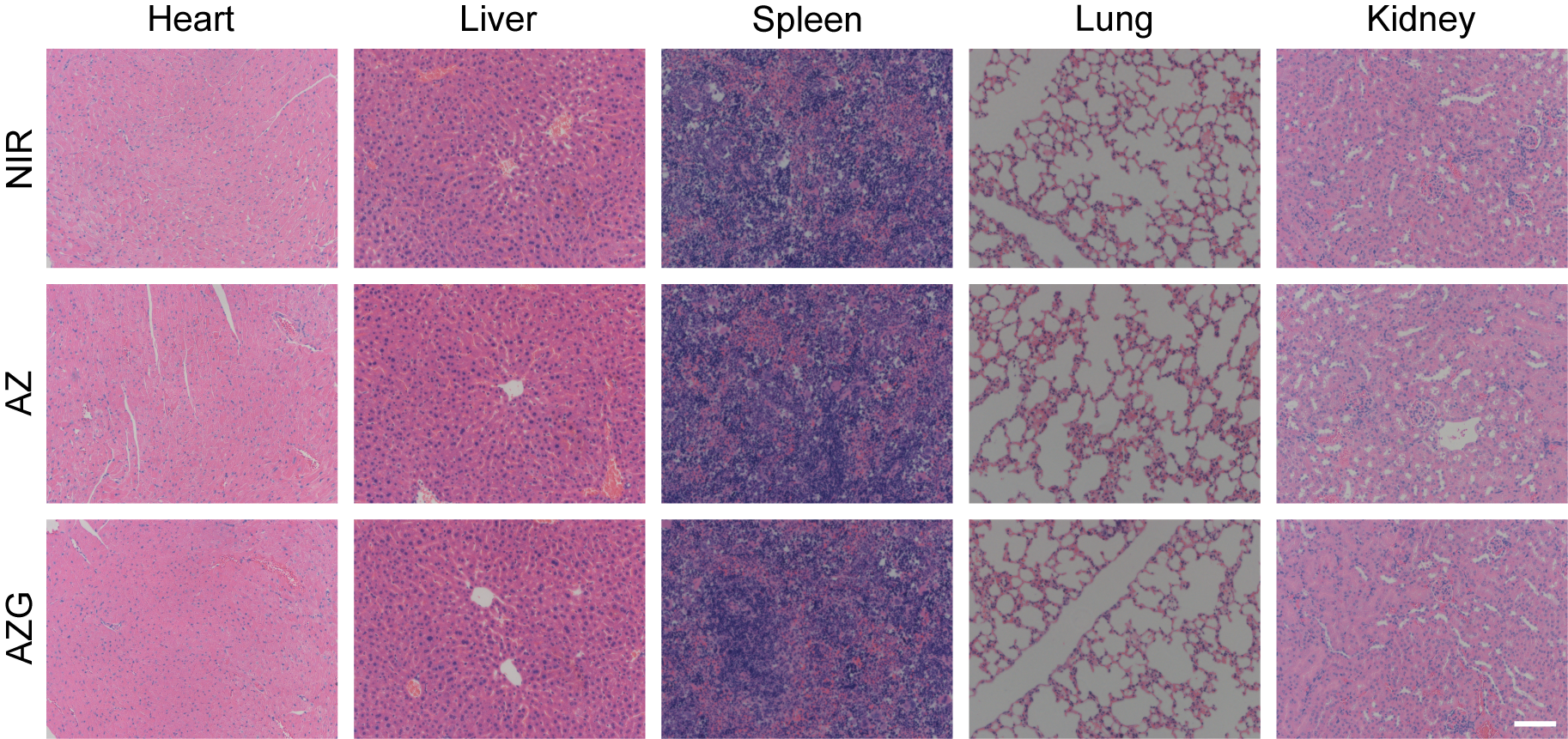


**Figure S17.** HE staining of the key organs slices in NIR, AZ and AZG groups (Scale bar is 100 μm).
